# The novel *GCK* variant p.Val455Leu associated with hyperinsulinism is susceptible to allosteric activation and is conducive to weight gain and the development of diabetes

**DOI:** 10.1007/s00125-021-05553-w

**Published:** 2021-09-16

**Authors:** Sara Langer, Rica Waterstradt, Georg Hillebrand, René Santer, Simone Baltrusch

**Affiliations:** 1 Institute of Medical Biochemistry and Molecular Biology, University Medicine Rostock, Rostock, Germany; 2 Department of Pediatrics, University Medical Center Eppendorf, Hamburg, Germany; 3 Present Address: Department of Pediatrics, Medical Center Itzehoe, Itzehoe, Germany; 4 Department Life, Light & Matter, University of Rostock, Rostock, Germany

**Keywords:** Congenital hyperinsulinism, Fructose 2,6-bisphosphatase, Glucokinase, Glucokinase regulatory protein, Glucose homeostasis, Mannoheptulose, RO-28-1675

## Abstract

**Aims/hypothesis:**

The mammalian enzyme glucokinase (GK), expressed predominantly in liver and pancreas, plays an essential role in carbohydrate metabolism. Monogenic GK disorders emphasise the role of GK in determining the blood glucose set point.

**Methods:**

A family with congenital hyperinsulinism (CHI) was examined for *GCK* gene variants by Sanger sequencing. A combined approach, involving kinetic analysis (also using GK activators and inhibitors), intracellular translocation assays, insulin secretion measurements and structural modelling, was used to investigate the novel variant compared with known variants.

**Results:**

We report on the novel gain-of-function *GCK* variant p.Val455Leu (V455L), inherited as an autosomal dominant trait in a German family with CHI and concomitant obesity (fasting blood glucose 2.1 mmol/l, BMI 45.0 kg/m^2^, HOMA-IR 1.5 in an adult female family member); one male family member developed type 2 diabetes until age 35 years (with fasting glucose 2.8–3.7 mmol/l, BMI 38.9 kg/m^2^, HOMA-IR 4.6). Kinetic characterisation of the V455L variant revealed a significant increase in glucose affinity (glucose concentration at which reaction rate is half its maximum rate [*S*_0.5_]: mutant 2.4 ± 0.3 mmol/l vs wild-type 7.6 ± 1.0 mmol/l), accompanied by a distinct additive susceptibility to both the endogenous activator fructose 2,6-bisphosphatase and the synthetic allosteric activator RO-28-1675. The effect of RO-28-1675 was more pronounced when compared with the previously known GK variants V455M and V455E. Binding to the inhibitor glucokinase regulatory protein was unimpaired for V455L and V455E but was reduced for V455M, whereas mannoheptulose inhibited all GK variants and the wild-type enzyme. Structural analyses suggested a role for residue 455 in rearrangements between the inactive and active conformations of GK and also in allosteric activation. Comparison with V455M and V455E and an overview of activating GK variants provided a context for the novel sequence aberration in terms of altered GK enzyme characteristics caused by single amino acid changes.

**Conclusion/interpretation:**

We provide new knowledge on the structure–function relationship of GK, with special emphasis on enzyme activation, potentially yielding fresh strategic insights into breaking the vicious circle of fluctuating blood glucose levels and the attendant risk of long-lasting metabolic changes in both CHI and type 2 diabetes.

**Graphical abstract:**

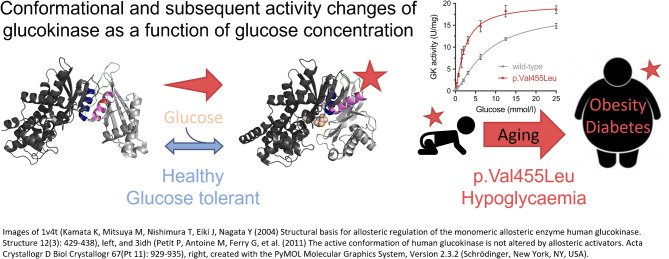

**Supplementary Information:**

The online version contains peer-reviewed but unedited supplementary material available at 10.1007/s00125-021-05553-w.

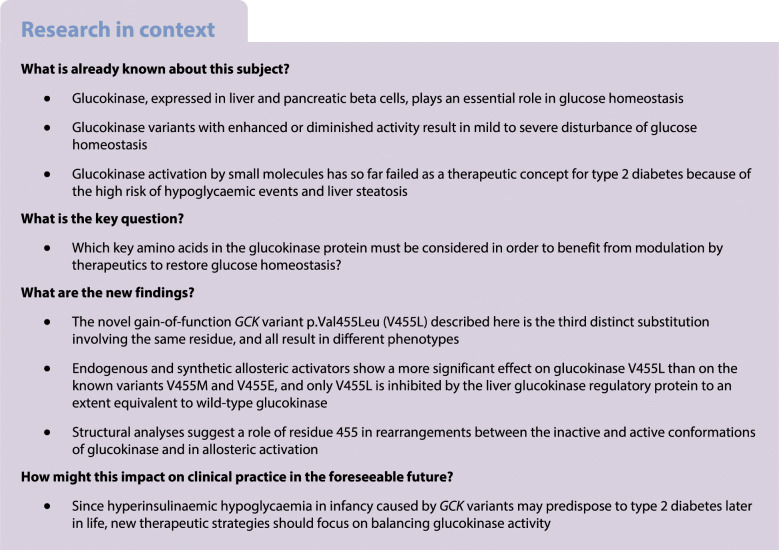



## Introduction

**Fig. 1:**
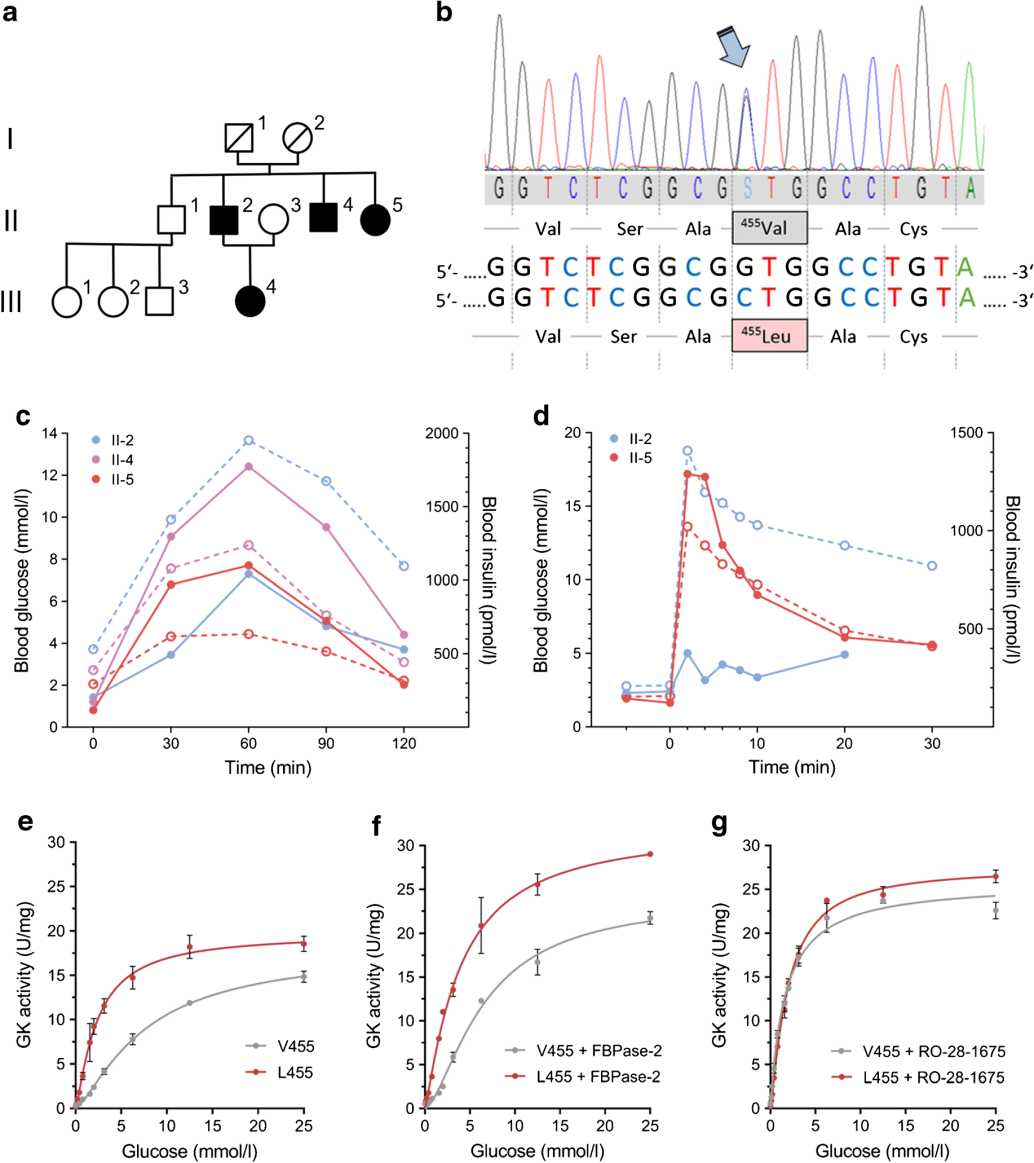
Novel *GCK* missense variant p.Val455Leu in a family with hyperinsulinism. (**a**) Pedigree of the affected family: black symbols indicate individuals diagnosed with hyperinsulinaemic hypoglycaemia of infancy. Male family members are represented by squares and female family members by circles. Roman numerals denote generations and Arabic numerals individuals within one generation, representing the birth order of siblings. (**b**) *GCK* sequencing identified heterozygosity for the novel variant NM_000162.3:c.1363G>C (p.Val455Leu) in the affected individuals. (**c**) Results of OGTT in individuals II-2, II-4 and II-5. For this test, 75 g glucose was given orally and blood glucose (white symbols, dashed lines) and insulin (filled symbols, solid lines) were measured every 30 min. (**d**) Results of IVGTT in individuals II-2 and II-5. A glucose bolus of 300 mg/kg was given and blood glucose (white symbols, dashed lines) and insulin (filled symbols, solid lines) were measured at indicated intervals. (**e**–**g**) Glucose-phosphorylating activity of 1 μg recombinant wild-type (V455) or L455 variant Dendra2-GK enzymes were measured (**e**) without any activating substances or after 25 min incubation with 3 μg of FBPase-2 (**f**) or 10 μmol/l RO-28-1675 (**g**). The graphs show fitted mean values ± SEM from five (wild-type) or three (L455) independent experiments. Non-linear regression was performed to compare allosteric sigmoidal or Michaelis–Menten model fitting. While the data for the wild-type enzyme treated with RO-28-1675 were better described by the Michaelis–Menten model, for all other curves, including those of the mutant, sigmoidal fitting was applied. The kinetic parameters calculated from the curves are listed in Table 1

The mammalian glucose-phosphorylating enzyme glucokinase (GK; hexokinase IV) plays a central role in glucose homeostasis. Most of our knowledge concerning GK function has been acquired from studies in liver (where about 99% of the body’s GK is found) and pancreatic beta cells but it is also expressed in pancreatic alpha cells and glucose-sensitive cells of the brain and the gut [1–6]. Kinetic features different from those of hexokinases I–III are crucial for GK to fulfil its role as a glucose sensor; namely, cooperative kinetics with regard to glucose, including an inflection point of about 3.5 mmol/l despite the fact that GK is monomeric, low affinity for glucose and a lack of feedback inhibition [6]. A large number of GK variants, the vast majority of which reduce its enzymatic activity, have been described in humans and their kinetic characterisation has emphasised the place of GK in determining the set point of glucose homeostasis [7]. Thus, activity-diminishing GK variants cause MODY2 or permanent neonatal diabetes mellitus (PNDM), whereas activating GK variants result in congenital hyperinsulinism (CHI). All naturally occurring GK mutation-mediated CHI (GK-CHI) variants described to date relate to 17 positions in the GK protein sequence. A further 19 activating GK mutants have been described in vitro, 12 of which are at residues where naturally occurring CHI mutations are hitherto unknown (electronic supplementary material [ESM] Fig. 1, ESM Table 1). Remarkably, some sequence aberrations that were predicted in a library search to result in GK activation [8] or that were engineered and kinetically characterised as activating [9, 10] have subsequently been found in humans, namely V91L, W99C, N180D, M197T, M197V and I211F, the last of which is non-congenital but somatic (ESM Table 1). In this study, we investigate the novel naturally occurring activating GK variant V455L, associated with CHI, and report results of meticulous kinetic and structural analyses. Residue 455 is part of the C-terminal helix α13, which is essential for both the cooperativity and the catalytic activity of the enzyme [11]. We systematically evaluate the kinetic properties of the novel variant V455L in comparison with those of the wild-type enzyme and previously described naturally occurring CHI and MODY2 mutants, respectively, V455M [12, 13] and V455E [14, 15]. The responses of the GK variants to the endogenous GK activator fructose 2,6-bisphosphatase (FBPase-2) [16], the allosteric activator RO-28-1675 [17] and the inhibitors glucokinase regulatory protein (GKRP) [18] and mannoheptulose [19] were also investigated, as well as the variants’ effect on insulin secretion. In addition, we summarise the activating *GCK* variants published to date, both naturally occurring and engineered (ESM Fig. 1).

## Methods

### Materials

Details are provided in ESM Methods.

### Participants and genetic analyses

RS and GH (together with J. Bergmann, K. Tsiakas and K. Ullrich from the Department of Pediatrics, University Medical Center Hamburg-Eppendorf, Hamburg, Germany and M. Witsch from the Department of Pediatrics, University Children Hospital Mannheim, Germany) detected the novel *GCK* variant in a German family; the variant is also known from a case report concerning a Polish family [20]. Our index case, a new-born female infant with a normal birthweight, presented at 2 weeks of age with symptomatic hypoglycaemia. Mild hyperinsulinism was detected with responsiveness to diazoxide. Hypoglycaemia in infancy was also documented in the father and two of his siblings. To elucidate the cause of the disease, sequence analyses and further investigations were performed in the female infant and family members. Genomic DNA was extracted from peripheral lymphocytes by standard methods and the coding regions of the ten exons including the intron–exon boundaries of the *GCK* gene were amplified by PCR. The products were purified and Sanger sequenced, and the results were compared with our reference sequence ENST00000403799 (NM_000162.3). The newly identified *GCK* variant has been submitted to the Leiden Open Variation Database (LOVD; https://www.lovd.nl, accessed 26 July 2021) (individual ID no. 00295607, DB-ID GCK_000213). Glucose tolerance tests were performed as described in ESM Methods. Written informed consent was obtained from all family members or parents mentioned, as appropriate and all investigations were done in accordance with the Declaration of Helsinki as revised in 2008.

### Plasmids and cDNA

All experiments were performed with the beta cell isoform of human GK. The GK-CHI mutants V455L (GK-L455) and V455M (GK-M455) [12] as well as the MODY2 mutant V455E (GK-E455) [14, 15, 21] were introduced into the vector pDendra2-C-GK wild-type, which is based on the mammalian expression vector pDendra2-C (Evrogen, Moscow, Russia) and encodes an N-terminal fusion of the fluorescent protein Dendra2 to GK [22]. Site-directed mutagenesis was performed with the QuikChange II Site-Directed Mutagenesis Kit (Agilent, Santa Clara, CA, USA) according to the manufacturer’s instructions. cDNAs of Dendra2-GK variants were amplified by PCR, introducing BamHI and NotI restriction sites, and subcloned into pGEX-6P-1 [23]. Mutagenesis and correct insertion of the full cDNAs were verified by sequence analysis. Rat liver bisphosphatase domain of 6-phosphofructo-2-kinase-2/FBPase-2 (residues 250–470) has previously been subcloned into the vector pGEX-6P-1 [24].

### Expression and purification of recombinant proteins

Dendra2-GK wild-type, -M455, -E455, -L455 and FBPase-2 were expressed as N-terminally glutathione *S*-transferase (GST) tagged fusion proteins in *Escherichia coli* BL21 at 30°C and were purified using Glutathione Sepharose 4B (GE Healthcare, Chalfont St Giles, UK) according to the manufacturer’s instructions, including enzymatic cleavage of the GST tag. Protein quality was checked by western blot analyses (ESM Fig. 2) using anti-GK antibody (Santa Cruz Biotechnology, Dallas, Texas, USA) as described previously [25].

### Hepatocyte isolation, culture and transfection

Experiments were performed as described previously [26, 27]. See also ESM Methods.

### GKRP immunoprecipitation from hepatocytes

GKRP from primary rat hepatocytes was isolated by immunoprecipitation using anti-GKRP antibody (Santa Cruz Biotechnology) non-covalently coupled to Dynabeads Protein G (Thermo Fisher Scientific, Waltham, MA, USA) according to the manufacturer’s instructions. Finally, the protein was eluted with 50 mmol/l glycine (pH 2.8). See ESM Methods for further details.

### Cell culture and transfection

MIN6 cells (originally provided by J.-I. Miyazaki [Osaka University Medical School, Osaka, Japan] and regularly checked on freedom from mycoplasm by Vendor GeM Classic Kit [Minerva Biolabs, Berlin, Germany]) were grown and transiently transfected using jetPEI (Polyplus, Illkirch, France). Overexpression of Dendra2-GK fusion protein was confirmed by western blot [25] and fluorescence microscopy analyses.

### GK enzyme activity

The glucose-phosphorylating activity of the recombinant Dendra2-GK variants, treated with FBPase-2, RO-28-1675 (Axon Medchem BV, Groningen, the Netherlands) or mannoheptulose (Glycoteam, Hamburg, Germany) where appropriate, was measured as described previously [28]. See also ESM Methods.

### Insulin secretion

Static insulin secretion was measured in MIN6 cells that were transiently transfected with pDendra2-GK wild-type, -M455, -E455 or -L455, as described previously [23].

### Fluorescence microscopy

Hepatocytes and MIN6 cells were seeded and transfected on MatTek dishes (MatTek Corporation, Ashland, MA, USA). Finally, live-cell imaging was performed using a cellR/Olympus IX81 inverted microscope system as described previously [23].

### Structural analysis

Structural images of human GK were prepared on the basis of protein data bank identification (PDB ID) 1v4s, 1v4t [29], 3idh [30] and 4dch [31] using the PyMOL Molecular Graphics System, Version 2.3.2 (Schrödinger, New York, NY, USA). See ESM Methods for further details.

### Data and statistical analysis

Statistical analyses were performed using the GraphPad Prism 8.1.1 analysis program (GraphPad Software, San Diego, CA, USA) and the results are presented as means ± SEM. Differences were examined using ANOVA/Bonferroni’s correction or Student’s *t* test, and values of *p* < 0.05 were considered to be statistically significant.

## Results

### Novel activating *GCK* missense variant p.Val455Leu

In the course of medical examinations conducted in a German family in order to elucidate the cause of persistent hyperinsulinaemic hypoglycaemia of infancy, sequencing of the *GCK* gene identified heterozygosity for the novel missense variant c.1363G>C, predicting an amino acid replacement p.Val455Leu. The affected family members were a new-born girl (III-4), her father (II-2) and two of his siblings (II-4 and II-5), suggesting a dominant trait (Fig. 1a,b). The girl became overweight during her first years of life (BMI 91st percentile) with persistently low blood glucose concentrations over the 6 years and 3 months of follow-up, HbA_1c_ of 22 mmol/mol (4.2%) (normal range: 26–46 mmol/mol [4.5–6.4%]) at that time, and no signs of insulin resistance (HOMA-IR 1.2, QUICKI 0.372). For the adult family members, a similar course in infancy was reported, with extreme appetite and development of massive obesity. An OGTT was performed in these affected adults (Fig. 1c): this verified impaired glucose tolerance in family member II-2, who had developed a BMI of 38.9 kg/m^2^ and type 2 diabetes with no autoantibodies detectable against GAD or against tyrosine phosphatase-related islet antigen 2 (IA-2) up to the time of this examination at age 35 years. The acute insulin response of individual II-2 was abolished at that time, while his fasting glucose levels were still low (2.8–3.7 mmol/l) and his HbA_1c_ was 39 mmol/mol (5.7%), with borderline fasting insulin levels (173.8–202.7 pmol/l [normal range: 18.8–181.0 pmol/l]) (Fig. 1d) and substantial signs of insulin resistance (HOMA-IR 4.6, QUICKI 0.305). Family member II-5 (also extremely obese with a BMI of 45.0 kg/m^2^) showed reduced fasting glucose level (2.1 mmol/l [normal range: 3.3–6.1 mmol/l]), with little increase after glucose intake, accompanied by inappropriately high insulin secretion (Fig. 1c). The IVGTT in individual II-5 revealed an existing acute insulin response, although secretion was again high (Fig. 1d). Her HbA_1c_ was 29 mmol/mol (4.8%); HOMA-IR and QUICKI were 1.5 and 0.360, respectively. The hyperinsulinaemic response of family member II-4, who was similarly obese, was intermediate between that of his siblings, as were indices of insulin resistance (HOMA-IR 2.9, QUICKI 0.326), although he showed less pronounced hypoglycaemia than his sister (Fig. 1c). He was not available for further investigations. The eldest brother (II-1; Fig. 1a) was healthy and of normal weight.

### Effects of the novel p.Val455Leu variant on GK kinetics

The effect of the novel variant GK-L455 on enzymatic activity was then studied with recombinant Dendra2-GK fusion proteins. Fusion to the photoconvertible fluorescent protein Dendra2 does not affect the kinetics of the GK enzyme [28] and allows direct comparability with cellular analyses. The GK-L455 mutant exhibited a distinct left shift of the glucose dependency curve, accounting for the increased enzymatic activity at physiological glucose concentrations (Fig. 1e). The half-maximal reaction rate of GK-L455 was achieved at a glucose concentration of 2.4 ± 0.3 mmol/l compared with 7.6 ± 1.0 mmol/l for the wild-type enzyme with similar turnover numbers; however, curve fitting still revealed a preference for the allosteric sigmoidal model over the Michaelis–Menten model, as for the wild-type enzyme, with similar Hill numbers (Table 1). Thus, the values for the wild-type are commensurate with the published data, which show a certain range of variation due to different protein purification methods and the high sensitivity of GK to oxidative stress [32]. Furthermore, although intrinsically of higher activity than wild-type GK, the L455 variant was markedly activated both by the endogenous GK binding partner FBPase-2 and by the allosteric GK activator (GKA) RO-28-1675 at physiological glucose concentrations (Fig. 1f,g, Table 1).

**Table 1:** Kinetic parameters of the activating GK-L455 mutant compared with the wild-type enzyme

Enzyme	*k*_cat_ (1/s)	*S*_0.5_ (mmol/l)	*h*	*K*_m_ (mmol/l)
GK wild-type				
No activation	23.8±1.5	7.6 ± 1.0	1.4 ± 0.1	–
+ FBPase-2	32.0±1.9*	6.6 ± 0.7	1.6 ± 0.1	–
+ RO-28-1675	34.5±1.0**	–	–	1.6 ± 0.2^‡^
GK-L455				
No activation	26.3±1.5	2.4 ± 0.3^††^	1.3 ± 0.2	–
+ FBPase-2	42.9±2.6**^†^	3.9 ± 0.5^†^	1.2 ± 0.1	–
+ RO-28-1675	36.7±1.1**	1.9 ± 0.1	1.3 ± 0.1	–

The parameters were calculated from the fitted curves shown in Fig. 1e–g (allosteric sigmoidal or, in the case of the wild-type enzyme with RO-28-1675 activation, Michaelis–Menten model) and are expressed as means ± SEM

Both Dendra2-GK variants were expressed recombinantly and analysed in the absence or presence of FBPase-2 or RO-28-1675

*p < 0.05 and **p < 0.01 compared with the same GK variant without activation; ^†^*p* < 0.05 and ^††^*p* < 0.01 compared with the respective parameter of wild-type GK; ^‡^*p* < 0.05 compared with the glucose concentration at half-maximal reaction rate (*S*_0.5_) of GK wild-type without activation (Student’s *t* test)

*h*, Hill number, *k*_cat_, enzymatic turnover number; *K*_m_, Michaelis–Menten constant; *S*_0.5_, concentration of the substrate glucose at half-maximal reaction rate

### Effects of RO-28-1675, FBPase-2 and mannoheptulose on GK-M455, -E455 and -L455 enzyme activity and measurements of thermostability

**Fig. 2:**
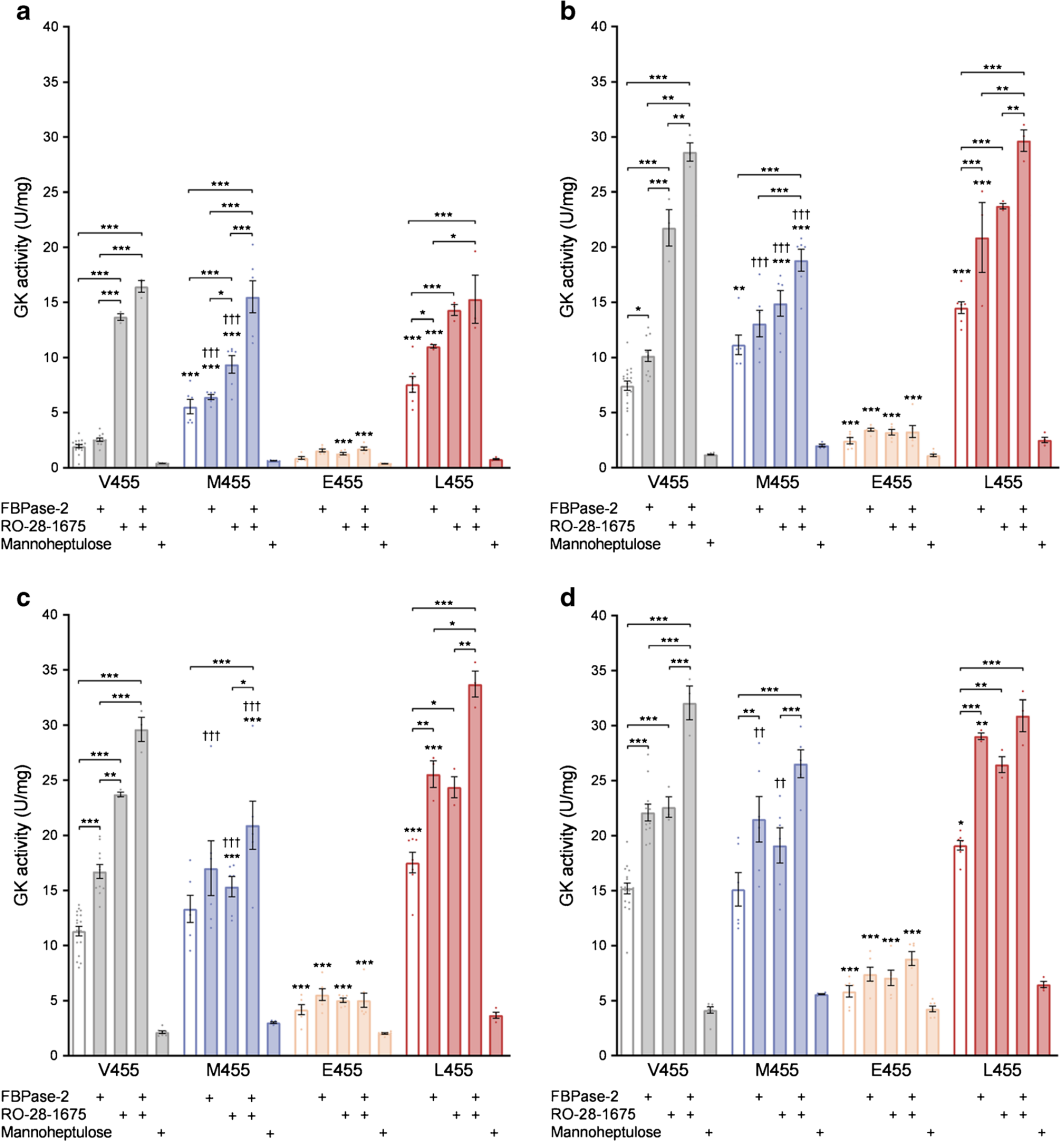
Response of recombinant Dendra2-GK wild-type (V455), -M455, -E455 and -L455 enzymes to endogenous and synthetic activators and to the competitive inhibitor mannoheptulose. Glucose-phosphorylating activity of the recombinant Dendra2-GK variants (1 μg or, when inhibited by mannoheptulose, 2 μg) were measured at 2 (**a**), 5 (**b**), 10 (**c**) or 25 mmol/l glucose (**d**) after 25 min incubation without supplements, with 3 μg recombinant FBPase-2, with 10 μmol/l RO-28-1675, with both activators combined, or with 10 mmol/l mannoheptulose. Data are shown as means ± SEM from four independent experiments. *p < 0.05, **p < 0.01 and ***p < 0.001 compared with wild-type GK, or, within one GK variant, as indicated by the brackets; ^††^*p* < 0.01 and ^†††^*p* < 0.001 compared with the activating mutation GK-L455; inhibition by mannoheptulose was significant for all GK variants analysed (*p* < 0.05) but for clarity symbols have been omitted (ANOVA/Bonferroni or, within the GK-E455 dataset, Student’s *t* test)

Subsequent detailed analysis of enzyme activity at four different glucose concentrations (2, 5, 10 and 25 mmol/l) combined with activating and inhibiting substances included two previously described GK mutants at position 455, namely the CHI variant GK-M455 [12, 13] and the MODY2 variant GK-E455 [14, 15] (Fig. 2). Glucose-phosphorylating activity was significantly reduced by the competitive inhibitor mannoheptulose for all GK mutants analysed, as for the wild-type enzyme. While the inherently low activity of the MODY2 mutant GK-E455 was enhanced by FBPase-2 in a proportion similar to the wild-type enzyme, this variant was only slightly activated by RO-28-1675. For both activating GK mutants (at 2 mmol/l glucose for GK-L455 half maximal effective concentration [EC_50_] = 3.97 ± 1.49 μmol/l and for GK-M455 EC_50_ = 7.19 ± 2.65 μmol/l), RO-28-1675 activation was also less pronounced than for the wild-type enzyme (at 2 mmol/l glucose EC_50_ = 0.88 ± 0.29 μmol/l), as reported previously for treatment of GK-M455 with a closely related GKA [33]. Interestingly, the activity of the novel variant GK-L455 was higher than that of the known GK-CHI variant GK-M455. This was particularly evident when the enzymes were treated with the activators FBPase-2 and/or RO-28-1675 (Fig. 2). Furthermore, similar to wild-type GK, both GK-M455 and GK-L455 were additively activated by FBPase-2 and RO-28-1675, as previously reported for the GK-CHI variants G68V and S64Y [28]. The thermal stability of all mutants analysed in this study was comparable with that of wild-type GK (ESM Fig. 3).

### Effect of GK variants -M455, -E455 and -L455 on insulin secretion in MIN6 cells

**Fig. 3:**
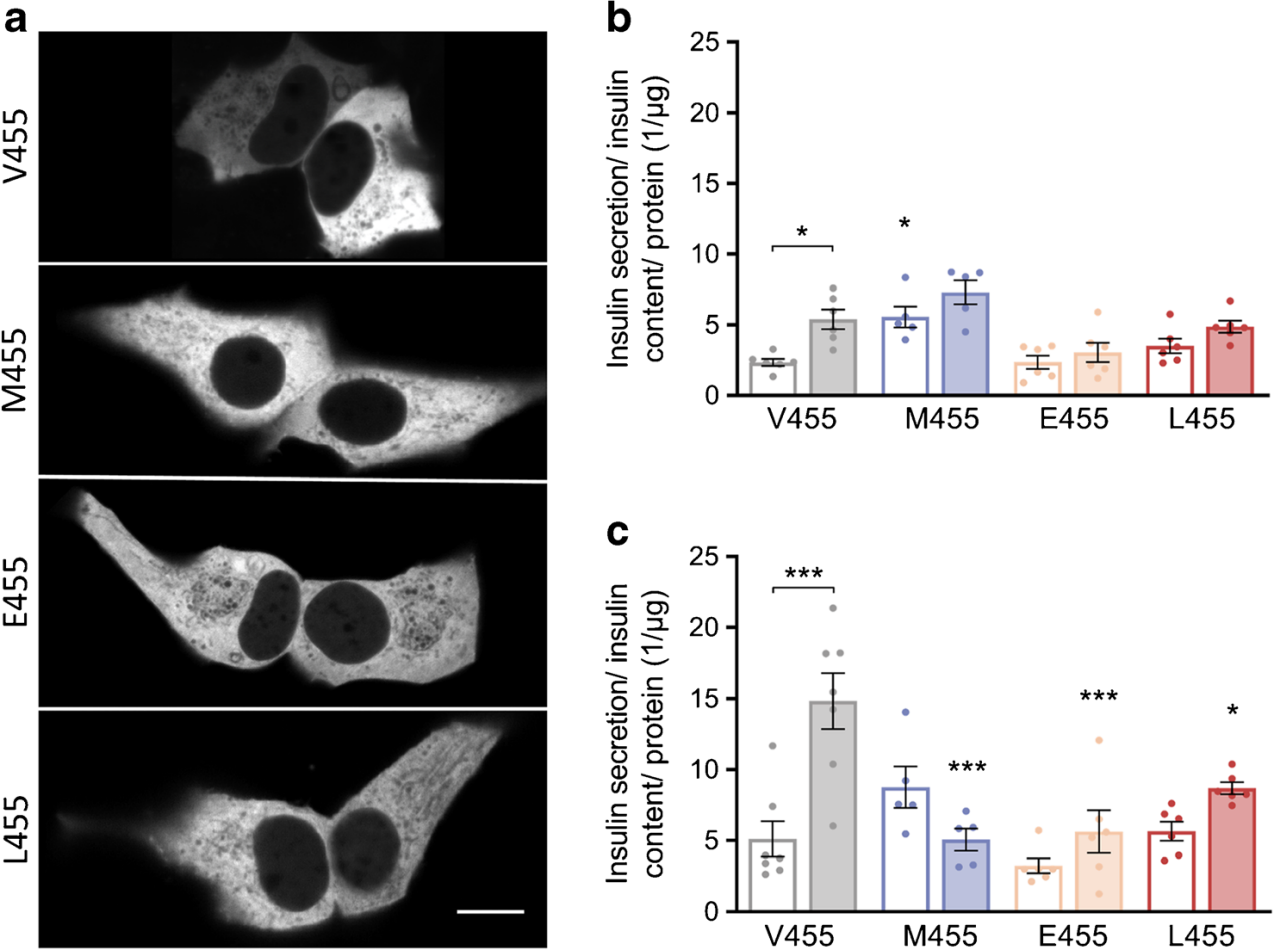
Intracellular localisation of Dendra2-GK variants and insulin secretion. (**a**) Representative images of insulin-secreting MIN6 cells transiently transfected with Dendra2-GK wild-type (V455) and mutants GK-M455, -E455 and -L455, showing homogeneous cytoplasmic distribution of Dendra2 fluorescence. Scale bar, 10 μm. (**b**, **c**) Static insulin secretion of MIN6 cells transiently overexpressing Dendra2-GK wild-type and -M455, -E455 or -L455 mutants. The cells were incubated in the absence of glucose for 1 h prior to stimulation with 3 mmol/l (white bars) or 10 mmol/l (filled bars) glucose for 1 h in the absence (**b**) or presence (**c**) of the activator RO-28-1675 (10 μmol/l). The insulin secretion was normalised to the insulin content before being normalised to the total protein content. Data are shown as means ± SEM from five independent experiments. *p < 0.05 and ***p < 0.001 compared with the wild-type enzyme, or, when indicated by brackets, compared with stimulation with 3 mmol/l glucose (ANOVA/Bonferroni)

The Dendra2-GK variants were transiently expressed in glucose-responsive MIN6 cells. All mutants, GK-M455, -E455 and -L455, were localised in the MIN6 cytosol (Fig. 3a), as was the wild-type enzyme [33]. Cells transfected with wild-type GK responded to stimulation with 10 mmol/l glucose with a significant increase in insulin secretion compared with stimulation with 3 mmol/l glucose. The increment in insulin secretion at high vs low glucose was less distinct for both the MODY2 and the CHI variants: for the former due to diminished secretion at 10 mmol/l glucose; for the latter due to increased basal insulin secretion, which was highest for GK-M455 (Fig. 3b). For all analysed GK variants, treatment with the GKA RO-28-1675 raised insulin secretion at low glucose to an amount similar to secretion at high glucose but without the GKA (Fig. 3c). With RO-28-1675, cells expressing GK-E455 or -L455 secreted more insulin at 10 mmol/l glucose compared with 3 mmol/l, although the extent of the increase was lower than for wild-type GK. Conversely, cells expressing GK-M455 secreted less insulin at 10 mmol/l glucose compared with 3 mmol/l when treated with RO-28-1675. However, after stimulation with 10 mmol/l glucose combined with RO-28-1675, cells transfected with wild-type GK secreted significantly more insulin than cells transfected with any of the mutants (Fig. 3c).

### Effects of GKRP on activity and intracellular localisation of GK-M455, -E455 and -L455

**Fig. 4:**
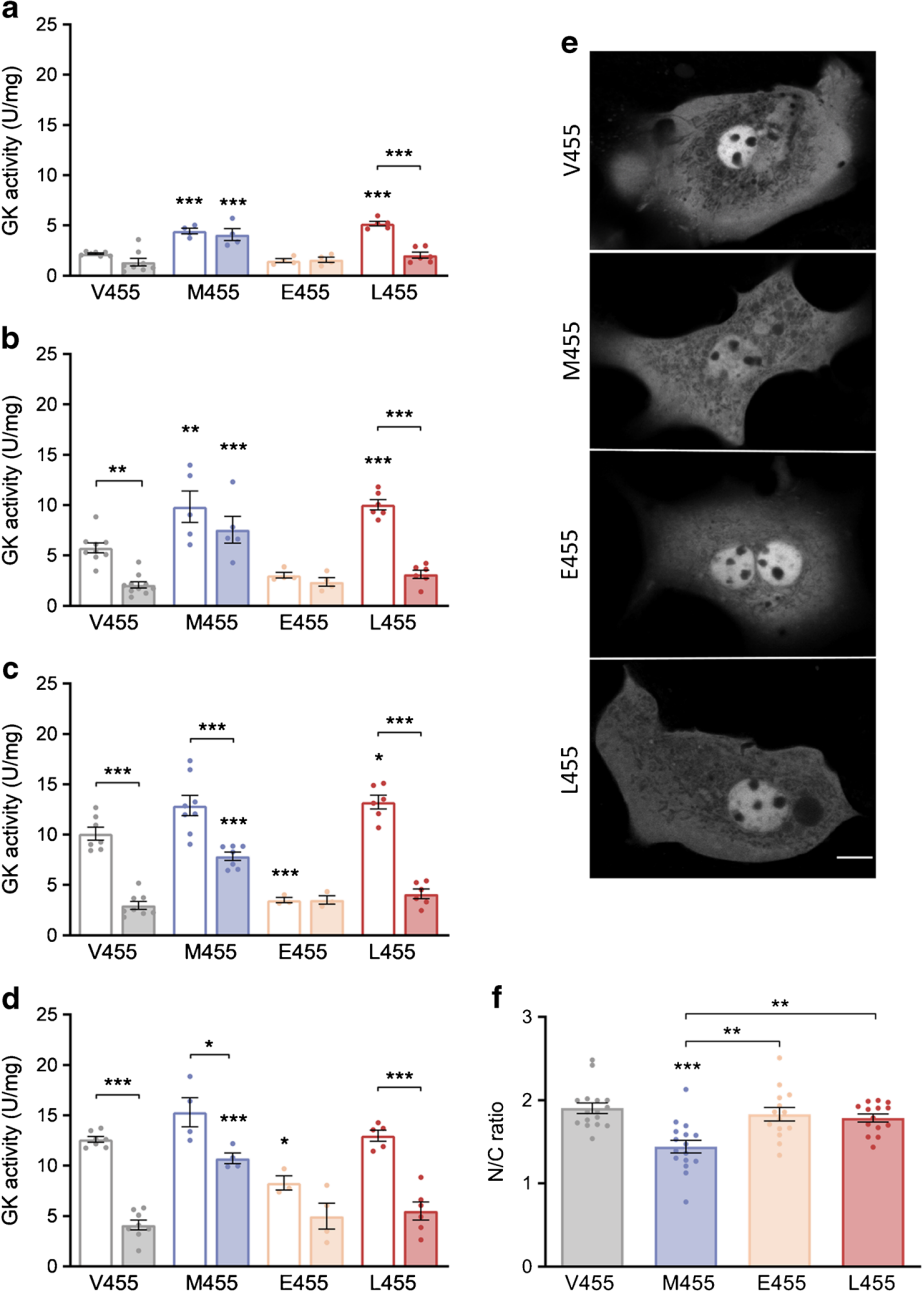
Effects of GKRP on enzyme activity and intracellular localisation of Dendra2-GK enzyme variants. (**a**–**d**) Glucose-phosphorylating activity of 2 μg recombinant Dendra2-GK wild-type (V455) and -M455, -E455 and -L455 enzymes were measured at 2 (**a**), 5 (**b**), 10 (**c**) or 25 mmol/l glucose (**d**) in the absence (white bars) or presence (filled bars) of 10 μg GKRP that was isolated from hepatocytes of male Wistar rats. Results shown are means ± SEM from four independent experiments. *p < 0.05, **p < 0.01 and ***p < 0.001 compared with the corresponding experiment with wild-type GK, or as indicated by the brackets, compared with enzyme activity without GKRP inhibition (ANOVA/Bonferroni). (**e**, **f**) Intracellular interaction of Dendra2-GK variants with GKRP in primary mouse hepatocytes. Representative fluorescence images of hepatocytes transiently transfected with Dendra2-GK wild-type and variants -M455, -E455 or -L455 and incubated with 25 mmol/l glucose (**e**). Scale bar, 10 μm. The nuclear/cytoplasmic (N/C) ratio of fluorescence intensity was calculated for hepatocytes transiently overexpressing Dendra2-GK wild-type, -M455, -E455 or -L455 (25 mmol/l glucose) (**f**). Data are shown as means ± SEM from a total of 14 cells (GK-E455, -L455) or 16 cells (GK wild-type, -M455) from four independent transfections. **p < 0.01 compared with the GK-M455 mutant; ***p < 0.001 compared with the wild-type enzyme (ANOVA/Bonferroni)

Inhibition of the recombinant Dendra2-GK enzymes by purified GKRP was studied at various glucose concentrations (Fig. 4a–d). Wild-type GK was inhibited by GKRP as expected, with a statistically significant effect emerging at glucose concentrations of 5 mmol/l and higher. GK-M455 activity was similarly reduced, although to a quantitatively lesser extent. Thus, while the activity of the wild-type enzyme was lowered by approximately 60–70% (at 5 mmol/l glucose or above), activity was reduced by only 20–40% for the GK-M455 mutant. The effect of GKRP on the GK-E455 variant was even smaller. This low-activity GK mutant was slightly but not significantly inhibited by GKRP only at the highest glucose concentration used (25 mmol/l). In contrast, the high-activity GK-L455 mutant was efficiently inhibited by GKRP, already showing a 60–70% reduction in phosphorylating activity at 2 mmol/l glucose (Fig. 4a). The Dendra2-GK variants were overexpressed in primary mouse hepatocytes to determine the intracellular localisation of the enzymes in the presence of endogenous GKRP (Fig. 4e,f). Whereas mutants GK-E455 and -L455 accumulated in the nucleus at 25 mmol/l glucose, with a nuclear/cytoplasmic ratio similar to the wild-type enzyme (1.83 ± 0.08 and 1.79 ± 0.05, respectively, compared with 1.91 ± 0.06), sequestration of the GK-M455 mutant in the nucleus was significantly reduced (nuclear/cytoplasmic ratio: 1.44 ± 0.08) (Fig. 4f).

### Effect of GK-M455, -E455 and -L455 variants on the structures of the inactive and active enzyme

**Fig. 5:**
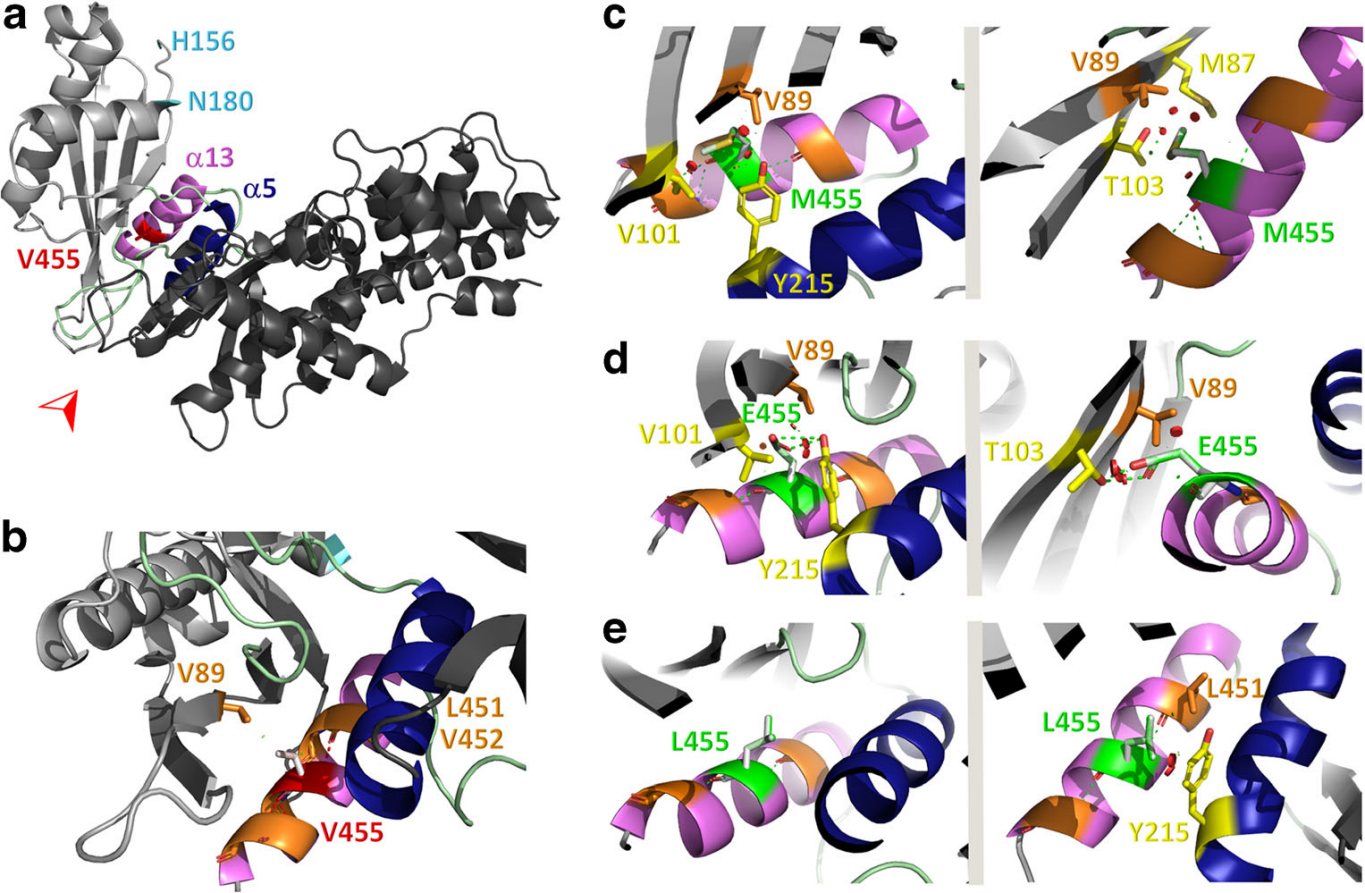
Predicted effects of Val-455 replacement by Met, Glu or Leu in a structural model of the super-open, enzymatically inactive GK conformation. (**a**) Within the super-open GK conformation (PDB ID 1v4t [29]) the large domain of the enzyme is shown in dark grey, the small domain in light grey, the interconnecting regions I–III in pale green, the C-terminal helix α13 in violet, and helix α5 in dark blue. Residues flanking a highly flexible structure with thus undefined conformation (His-156, Asn-180) are coloured light blue to distinguish them from the termini of the enzyme. Residue Val-455 is shown as a stick model and highlighted in red. The viewing direction for the details in (**b**–**e**) is indicated by the red arrowhead. (**b**) The enlarged section of the allosteric site includes polar contacts of residue 455 (dashed lines) as well as minor (green discs) or major (red discs) steric overlap with neighbouring amino acids. Neighbouring amino acids with contacts to Val-455 are coloured orange. Those contact residues whose interactions exceed the α-helical backbone hydrogen bonds are named (also in **c**–**e**). Within amino acids shown as stick models, oxygen atoms are coloured red and nitrogen atoms blue. (**c**) Mutation of Val-455 to methionine, with the mutated residue shown in green and the sulphur atom in yellow. Two possible rotamers are presented for illustrative purposes. Polar and steric contacts are shown as before, with amino acids interacting with the mutated (but not with the wild-type) residue coloured yellow. (**d**) Mutation of Val-455 to glutamate (colour scheme as before). (**e**) Mutation of Val-455 to leucine (colour scheme as before)

**Fig. 6:**
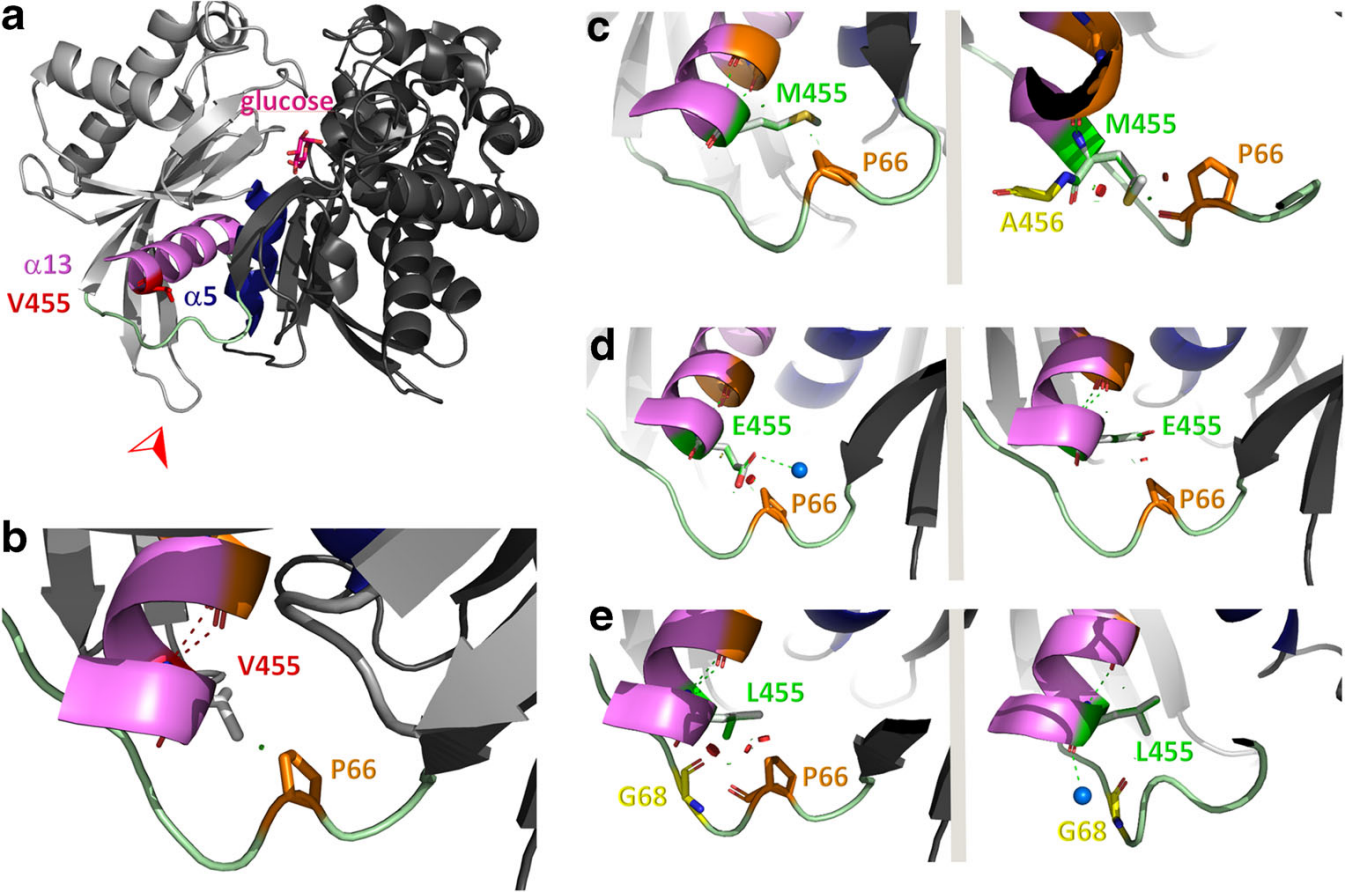
Predicted effects of Val-455 replacement by Met, Glu or Leu in a structural model of the closed, enzymatically active GK conformation. (**a**) Within the closed, glucose-bound GK conformation (PDB ID 3idh [30]) the large domain of the enzyme is shown in dark grey, the small domain in light grey, the interconnecting regions I–III in pale green, the C-terminal helix α13 in violet, helix α5 in dark blue, and the glucose molecule in magenta. Residue Val-455 is shown as a stick model and highlighted in red. The viewing direction for the details in (**b**–**e**) is indicated by the red arrowhead. (**b**) The enlarged section of the allosteric site includes polar contacts of residue 455 (dashed lines) as well as minor (green discs) or major (red discs) steric overlap with neighbouring amino acids. Neighbouring amino acids with contacts to Val-455 are coloured orange. Those contact residues whose interactions exceed the α-helical backbone hydrogen bonds are named (also in **c**–**e**). Within amino acids shown as stick models, oxygen atoms are coloured red and nitrogen atoms blue. (**c**) Mutation of Val-455 to methionine, with the mutated residue shown in green and the sulphur atom in yellow. Two possible rotamers are presented for illustrative purposes. Polar and steric contacts are shown as before, with amino acids interacting with the mutated (but not with the wild-type) residue coloured yellow. (**d**) Mutation of Val-455 to glutamate (colour scheme as before). The marine blue ball represents a water molecule. (**e**) Mutation of Val-455 to leucine (colour scheme as before)

**Fig. 7:**
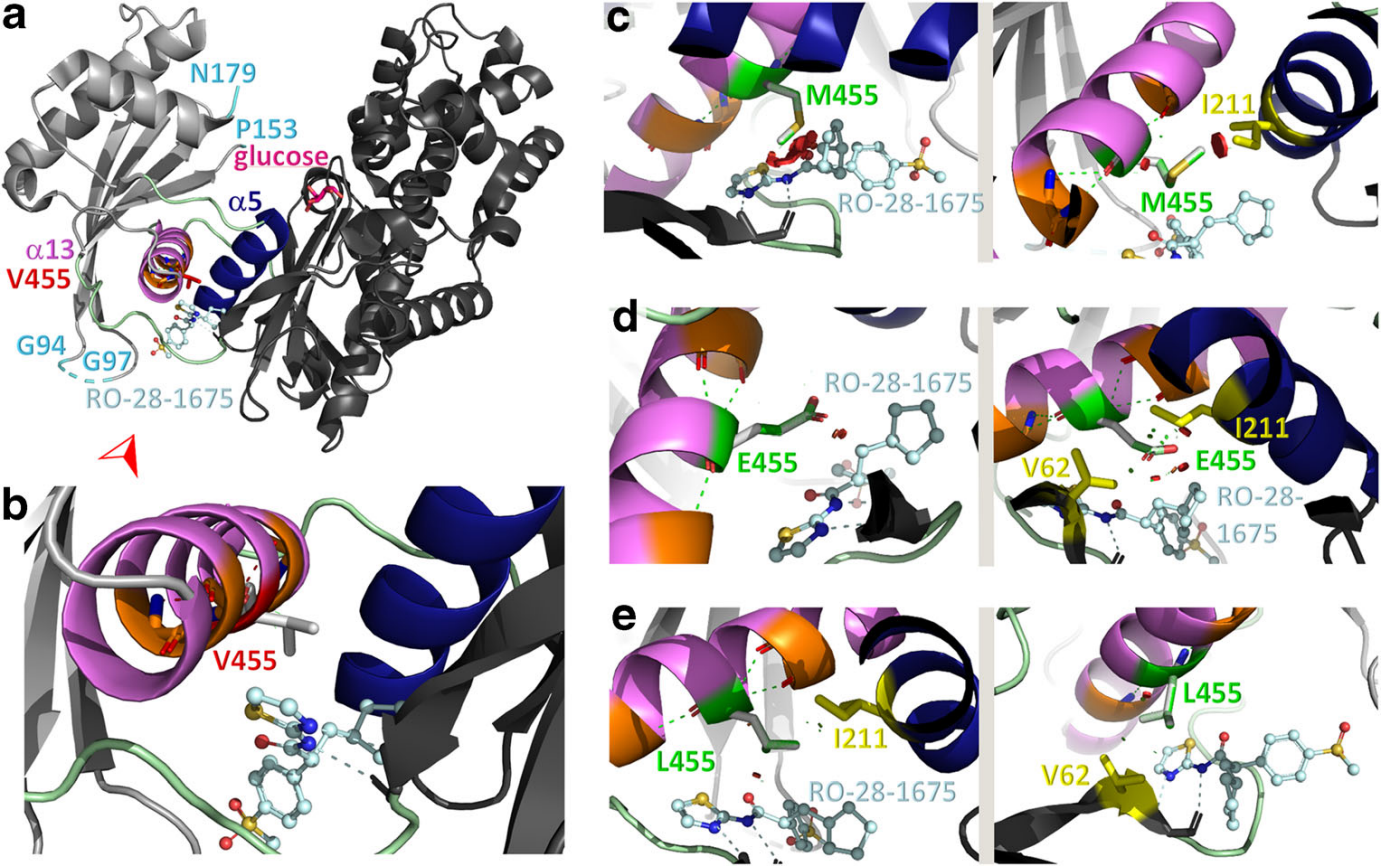
Predicted effects of Val-455 replacement by Met, Glu or Leu in a structural model of the active wide-open GK-RO-28-1675 complex. (**a**) Within the GKA-bound wide-open, glucose-bound GK conformation (PDB ID 4dch [31]) the large domain of the enzyme is shown in dark grey, the small domain in light grey, the interconnecting regions I–III in pale green, the C-terminal helix α13 in violet, helix α5 in dark blue, the GKA RO-28-1675 as a ball-and-stick model (blue-white; with oxygen atoms in red, nitrogen atoms in blue, and sulphur atoms in yellow) and the glucose molecule in magenta. Residues flanking highly flexible structures with thus undefined conformation (Gly-94, Gly-97, Pro-153, Asn-179) are coloured light blue to distinguish them from the termini of the enzyme. Residue Val-455 is shown as a stick model and highlighted in red. The viewing direction for the details in (**b**–**e**) is indicated by the red arrowhead. (**b**) The enlarged section of the allosteric site includes polar contacts of residue 455 and of RO-28-1675 (dashed lines). Neighbouring amino acids with contacts to Val-455 are coloured orange. Within amino acids shown as stick models, oxygen atoms are coloured red and nitrogen atoms blue. (**c**) Mutation of Val-455 to methionine, with the mutated residue shown in green and the sulphur atom in yellow. Two possible rotamers are presented for illustrative purposes. Polar contacts are shown as before and steric overlap as green and red discs. Amino acids interacting with the mutated (but not with the wild-type) residue are coloured yellow rather than orange. Those contact residues whose interactions exceed the α-helical backbone hydrogen bonds are named and, similarly, the GKA is named only when in contact with the mutated residue (also in **d**, **e**). (**d**) Mutation of Val-455 to glutamate (colour scheme as before). (**e**) Mutation of Val-455 to leucine (colour scheme as before)

Structural models of the GK super-open (1v4t), closed (3idh) and active wide-open (4cdh) conformations were analysed with regard to interactions of residue 455. In the super-open, inactive conformation of GK, the wild-type residue V455 makes hydrophobic contacts with side chains of a β-sheet cluster within the small domain of the enzyme, specifically V89 and V101 of sheets β3 and β4 (Fig. 5a,b). Due to the structural rearrangements during the transition between the super-open and the closed conformations, in the active enzyme the V455 side chain is no longer oriented towards the small GK domain but towards the allosteric site (Figs 6a,b, 7a,b). In the inactive GK conformation, mutation of valine to methionine will disturb the hydrophobic contacts between residue 455 and sheets β3 and β4 due to the larger space requirement of the methionine side chain (Fig. 5c). Additionally, collision of the methionine side chain with helix α5 (Y215) is possible (Fig. 5c). Conversely, mutation to glutamate will stabilise the super-open GK conformation due to the formation of hydrogen bonds either to helix α5 (Y215) or the small domain β-sheet cluster (T103) (Fig. 5d). Apparently, the new variant L455 can be introduced into the super-open GK structure without any disturbances but it may also collide with the Y215 side chain (Fig. 5e). Within the closed, glucose-bound GK conformation, all possible contacts of the 455 variants affect a loop region (interconnecting region I) that is part of the allosteric site of the enzyme (Fig. 6). Here, the larger side chains of the variants GK-M455, -E455 and -L455 evoke only minor steric conflicts, which nonetheless potentially alter the conformation of the loop within the active enzyme. In the active wide-open GK conformation (included in the analysis to study the environment of residue 455 in the GK-glucose-RO-28-1675 complex) the GKA is positioned between residue 455 and the loop structure of the allosteric site (Fig. 7). Replacement of valine by methionine will produce severe steric conflicts between the methionine side chain and either the GKA or residue I211 of helix α5 (Fig. 7c). Similar interferences, although less pronounced, are predicted when valine is substituted by glutamate (Fig. 7d). Conversely, the leucine side chain at position 455 will not impede binding of RO-28-1675, and even stabilisation of the complex due to hydrophobic interactions with either I211 (α5) or V62 (interconnecting loop) seems plausible (Fig. 7e).

## Discussion

The present study functionally characterises the activating *GCK* variant p.Val455Leu, recently documented in two European families with CHI, and places it in the context of previously identified sequence aberrations at the same site of the GK protein. Among the genetic mechanisms linked to CHI, inactivating variants of *ABCC8* and *KCNJ11* (encoding the two subunits of the ATP-sensitive potassium channels in the pancreatic beta cell) are most prevalent [34] but *GCK* gain-of-function mutants have been described in an increasing number of individuals since 1998 (ESM Table 1). The high heterogeneity of the clinical phenotype within people who have been diagnosed with GK-CHI is especially noteworthy. It ranges from severe symptoms from the first day of life, associated with mental retardation, epilepsy and a substantially reduced life expectancy [35], through to mild or even asymptomatic forms, occasionally diagnosed incidentally as GK-CHI in the context of genetic testing of family members. Diagnosis may also be complicated by insulin levels not always being elevated in hypoglycaemia, pointing to the impact of liver glucose disposal, which is also determined by GK activity, on glucose homeostasis [36]. Furthermore, there is no clear correlation between the severity of the clinical phenotype and the in vitro extent of activation of the mutated GK enzyme [37].

The novel variant p.Val455Leu affects the allosteric region of the GK protein where most of the currently known activating mutations are clustered. This binding site of the synthetic GKAs lies on the outer side of the GK hinge region, remote from the substrate-binding catalytic cleft, and is absent in the enzymatically inactive conformation [29]. Specifically, residue 455 is located within the C-terminal helix α13, which plays an important role in catalysis and cooperativity [11]. In the glucose-bound, closed GK conformation helix α13 is situated in the inner layer of the small domain of the enzyme at the domain interface, in a perpendicular orientation relative to helix α5 of the large domain [29]. During transition to the super-open state (a conformation of GK that does not exist within the other hexokinases) helix α13 is completely released from the small domain in a process that necessitates major structural rearrangements. As the catalytic turnover (involving the closed and an open conformation) is faster than the equilibration between the open and the super-open conformation, a slow reaction cycle at low glucose concentration contrasts with a fast reaction cycle; this combination results in sigmoidicity [29]. This structural flexibility includes rotation of helix α13 around its own axis and entails residue V455 being part of the allosteric site of GK in the closed, active conformation but being oriented towards the small domain of the enzyme in the super-open, inactive conformation. A feature V455L shares with several other activating GK variants is the addition of a longer, hydrophobic side chain to the allosteric site, thereby presumably stabilising the active conformation. Although the structural analyses indicate that the inhibitory effect of the MODY2 variant V455E results mainly from stabilisation of the super-open enzyme conformation via hydrogen bond formation, it cannot be excluded that electrostatic repulsion within the closed conformation (specifically between E455 and E67 of the flexible loop structure) contributes to the phenotype.

Apart from the direct effects of single amino acid substitutions on GK kinetics, possible in vivo effects of the variants on post-translational GK regulation must be considered. The binding site of the inhibitor GKRP has been shown to include regions of GK that, in the active conformation, are close to the allosteric site [38]. The effect of GKRP inhibition is more readily detectable under conditions where the initial value for the uninhibited activity of GK is high. This probably explains why the MODY2 variant GK-E455 is not affected by incubation with GKRP at glucose concentrations below 25 mmol/l and why the effect on the CHI variant GK-L455 is evident even at 2 mmol/l glucose. Indeed, the intracellular localisation of GK-E455 and -L455 in mouse hepatocytes is similar to that of the wild-type enzyme, indicating that these mutations do not significantly alter the interaction with GKRP. The GKRP binding conformation of GK closely resembles the super-open conformation [38]. Structural analyses have consistently indicated that the leucine side chain disturbs this conformation only marginally if at all and that the glutamate residue even stabilises it. In contrast, the methionine side chain prevents proper formation of the super-open conformation, possibly explaining the observed reduction in GKRP binding and inhibition of GK-M455. It remains unclear whether the reduced interaction is accompanied by increased glucose-phosphorylating capacity of the liver due to diminished inhibition of GK by GKRP or instead by decreased phosphorylating capacity due to enhanced liver GK protein degradation [39]. Decreased glucose clearance by the liver will result in increased insulin secretion to reach the blood glucose set point, and vice versa.

In the present study, one affected family member (II-2) with documented hypoglycaemia in infancy had developed type 2 diabetes by the time of examination (without previous pancreatectomy), as has been reported in three pedigrees for carriers of the activating *GCK* variants p.Val455Met, p.Gly68Val or p.Thr103Ser, respectively [12, 40–42]. Diabetes was diagnosed quite early (28–48 years of age) in the individuals carrying the *GCK* variants p.Val455Leu, p.Val455Met and p.Gly68Val. It has been reported previously that gain-of-function variants of GK are able to mediate beta cell toxicity by inducing either necrosis [43] or apoptosis [41, 44], with the involvement of DNA damage and both oxidative and endoplasmic reticulum stress [41, 45]. Because pedigrees of GK-CHI were first investigated two decades ago, increasing incidences of beta cell failure and type 2 diabetes are presumably inevitable. Evidently, the in vivo effects of the GK variants involve the entire network of GK-expressing tissues, protein-protein interaction partners of GK such as GKRP and FBPase-2, and conceivably a hitherto unidentified endogenous ligand to the allosteric site. Recent studies have indicated that beta cell-specific overexpression of the *GCK* variant p.Ala454_Val455insAla in mice is not sufficient to provoke weight gain [45]. Future research is necessary to investigate whether increased hepatic lipogenesis (analogous to the phenotype observed in individuals with GKRP variants showing reduced GK inhibition and thus enhanced GK activity [46]) contributes to obesity. Serum triacylglycerol levels were at least very low in family members II-5 and III-4, suggesting excessive uptake by adipose tissue due to hyperinsulinaemia, but were elevated in the diabetic individual II-2 (ESM Table 2). Furthermore, fat gain might be an additional protective mechanism used by the body to prevent a rapid postprandial decline in blood glucose [47]. In view of their rapid glucose clearance and findings indicating that, in contrast to other physiological responses to hypoglycaemia, feelings of hunger do not adapt to repeated occurrence of hypoglycaemic states [48], individuals with GK-CHI are presumably especially prone to such a metabolic vicious cycle. Indeed, a BMI increase around the time of puberty or being overweight in general have also been reported for carriers of the variant p.Val455Met [12, 13, 41] and other GK-CHI variants (denoted by ^‡^ in ESM Table 1).

In conclusion, in addition to characterising the novel activating *GCK* variant p.Val455Leu, we have updated knowledge concerning GK structure–function relationships, with special emphasis on enzyme activation.

## Supplementary Information

ESM (PDF 1.46 mb)

## Data Availability

The data that support the findings of this study are openly available in LOVD at https://databases.lovd.nl/shared/variants/GCK?search_position_c_start=1363&search_position_c_start_intron=0&search_position_c_end=1363&search_position_c_end_intron=0&search_vot_clean_dna_change=%3D%22c.1363G%3EC%22&search_transcriptid=00008409, within the article, or on request from the corresponding author.

## Abbreviations

CHI: Congenital hyperinsulinism
EC_50_: Half maximal effective concentration
FBPase-2: Fructose 2,6-bisphosphatase
GK: Glucokinase
GKA: Allosteric GK activator
GK-CHI: GK mutation-mediated CHI
GKRP: GK regulatory protein
GST: Glutathione *S*-transferase
PDB ID: Protein data bank identification
PNDM: Permanent neonatal diabetes mellitus

## Declarations

### Authors’ relationships and activities

The authors declare that there are no relationships or activities that might bias, or be perceived to bias, their work.
