# Supplementary material for: The novel GCK variant p.Val455Leu associated with hyperinsulinism is susceptible to allosteric activation and is conducive to weight gain and the development of diabetes
Source: Diabetologia. 2021 Sep 16;64(12):2687–700. doi: 10.1007/s00125-021-05553-w (PMC8563668; doi:10.1007/s00125-021-05553-w)
Supplement: Supplementary file 1 — (PDF 1.46 mb) [file 125_2021_5553_MOESM1_ESM.pdf]

Electronic supplementary material

The novel activating *GCK* variant p.Val455Leu is susceptible to allosteric activation and is conducive to weight gain and the development of diabetes

Sara Langer, Rica Waterstradt, Georg Hillebrand, René Santer and Simone Baltrusch

ESM Methods

Materials

RO-28-1675 was purchased from Axon Medchem BV, Groningen, The Netherlands, and mannoheptulose from Glycoteam, Hamburg, Germany. All other chemicals were bought from Merck KGaA, Darmstadt, Germany. Restriction enzymes, oligonucleotides, Bradford assay, Dynabeads Protein G and tissue culture equipment were purchased from Thermo Fischer Scientific, MA, USA. QuikChange II Site-Directed Mutagenesis Kit was bought from Agilent, Santa Clara, CA, USA, and GKRP (GCKR, G-6) antibody (sc-74544) and GK (GCK, G-6) antibody (sc-17819) from Santa Cruz Biotechnology, Dallas, Texas, USA. The GST recombinant protein expression system was obtained from GE Healthcare, Chalfont St Giles, UK. Cell culture dishes were bought from SARSTEDT, Nümbrecht, Germany, and glass-bottomed six-well microplates from MatTek Corporation, Ashland, MA, USA. The insulin ELISA was purchased from Mercodia, Uppsala, Sweden.

Oral and intravenous glucose tolerance tests

Oral glucose tolerance tests (OGTTs) were performed in three adult family members after an overnight fast. Blood samples for glucose and insulin determinations were taken immediately before ingestion of 75 g glucose (25% (w/v) solution in H_2O) and thereafter at 30 min intervals.

Intravenous glucose tolerance tests (IVGTTs) were performed in two adult family members with an intravenous injection of 300 mg glucose per kg body weight (20% (w/v) solution in H_2O). Blood samples for glucose and insulin determinations were taken immediately before glucose injection, on completion of the injection 5 min after the first blood sample (time zero), and then after 2, 4, 6, 8, 10, 20, and 30 min. Indices of insulin resistance (HOMA-IR, QUICKI) were determined, as previously described, with reference ranges of 1.57±0.87 and 0.366±0.029, respectively [1, 2].

Hepatocyte isolation, culture, and transfection

Primary hepatocytes were isolated from male Wistar rats as described previously [3] and stored at -80°C for GKRP isolation. Primary hepatocytes isolated from male C57BL/6J mice (The Jackson Laboratory, USA; https://www.jax.org/strain/000664) were cultured and transiently transfected with jetPEI-Hepatocyte (Polyplus, Illkirch, France) as described previously [4] for intracellular localisation studies. These procedures were conducted in accordance with the German Animal Welfare Act 2006 (last amended 2014) and were approved by the State Department of Agriculture, Food Safety and Fisheries, Mecklenburg-Vorpommern (LALLF M-V).

GKRP immunoprecipitation from hepatocytes

The supernatant of 50 µl Dynabeads Protein G (Thermo Fisher Scientific) was removed. The beads were mixed with 200 µl antibody solution (5 µg anti-GKRP antibody (Santa Cruz Biotechnology) in 200 µl PBS containing 0.01% (v/v) Tween-20), incubated on a roller for 2 h at 4°C, and the supernatant was removed. Then 5×10^7 hepatocytes were resuspended in 2 ml PBS (4°C), homogenised by sonication (5 min), and insoluble material was pelleted by centrifugation (7,000 g, 10 min, 4°C). Subsequently, 150 µl hepatocyte lysate and 150 µl PBS/0.05% Tween-20 were added to the prepared beads and incubated on a roller for 2 h at

4°C. The beads were washed twice with 200 µl PBS each and were resuspended in 100 µl PBS, transferred into a fresh reaction tube, and the supernatant was removed. The precipitated GKRP protein was eluted by addition of 20 µl 50 mmol/l glycine (pH 2.8), careful mixing by pipetting, and incubation on a roller for 2 min at 4°C. The eluate was transferred into a fresh tube and 20 µl 1 M TRIS (pH 7.5) were added. These elution steps were repeated twice, and the eluates were stored at -20°C.

GK enzyme activity

For GKRP-inhibition studies 2 µg Dendra2-GK protein were incubated in assay buffer for 25 min in the absence or presence of 10 µg isolated GKRP + 1 mmol/l fructose-6-phosphate (Merck KGaA, Darmstadt, Germany) prior to glucose addition. For determination of EC_{50} values of RO-28-1675 1 µg of recombinant Dendra2-GK wild-type, -M455, or -L455 enzymes were incubated in reaction buffer for 25 min at 37°C with increasing concentrations (0.3, 0.5, 1, 3, 5, 10, 20, 30 µmol/l) of RO-28-1675 [5] prior to measurement at 2 mmol/l glucose. Finally, EC_{50} values were calculated using [Agonist] vs. response (three parameters) dose-response stimulation model of the GraphPad Prism 8.1.1 analysis program (GraphPad Software, San Diego, CA, USA).

Western blot analyses

5 µg of purified recombinant pDendra2-GK wild-type, -M455, -E455 or -L455 protein were separated by SDS-PAGE and blotted onto Roti Fluoro PVDF membrane (Roth, Karlsruhe, Germany). All steps (including dilution of the antibodies) were carried out in Odyssey blocking buffer (LI-COR, Lincoln, NE, USA) diluted 1:3 in PBS. Membranes were incubated for 1 h at room temperature with the GK-antibody (1:500). Immunoreactive bands were visualized using the anti-mouse fluorescence-labelled secondary antibody IRDye 680 CW (1:5000) via the Odyssey imaging system (LI-COR).

Structural analysis – modelling of amino acid mutations

Modelling of the structures of GK variants p.Val455Met, p.Val455Glu, and p.Val455Leu was conducted with PyMOL Mutagenesis Wizard. The side chain orientation with minimal steric clashing within the protein structure was selected from the "Backbone Dependent Rotamers" library [6]. Subsequently, the PyMOL clean command was used to move atoms within a radius of 5 Å including residue 455 to their positions of lowest local energy. Beginning from this calculated structure, another rotamer with minimal steric clashing was selected and another clean-up performed to consider various possibilities for the prediction.

References ESM Methods

[1] Hrebicek J, Janout V, Malincikova J, Horakova D, Cizek L (2002) Detection of insulin resistance by simple quantitative insulin sensitivity check index QUICKI for epidemiological assessment and prevention. J Clin Endocrinol Metab 87(1): 144-147. 10.1210/jcem.87.1.8292
[2] Matthews DR, Hosker JP, Rudenski AS, Naylor BA, Treacher DF, Turner RC (1985) Homeostasis model assessment: insulin resistance and beta-cell function from fasting plasma glucose and insulin concentrations in man. Diabetologia 28(7): 412-419. 10.1007/BF00280883
[3] Baltrusch S, Francini F, Lenzen S, Tiedge M (2005) Interaction of glucokinase with the liver regulatory protein is conferred by leucine-asparagine motifs of the enzyme. Diabetes 54(10): 2829-2837
[4] Rees MG, Wincovitch S, Schultz J, et al. (2012) Cellular characterisation of the GCKR P446L variant associated with type 2 diabetes risk. Diabetologia 55(1): 114-122. 10.1007/s00125-011-2348-5
[5] Grimsby J, Sarabu R, Corbett WL, et al. (2003) Allosteric activators of glucokinase: potential role in diabetes therapy. Science 301(5631): 370-373. 10.1126/science.1084073
[6] Dunbrack RL, Jr., Karplus M (1993) Backbone-dependent rotamer library for proteins. Application to side-chain prediction. Journal of molecular biology 230(2): 543-574. 10.1006/jmbi.1993.1170

ESM Table 1

Variant	References
V62L	Gloyn et al., 2005
V62M*	Gloyn et al., 2005; Zelent et al., 2011
S64F	Pal & Miller, 2009
S64P	Pal & Miller, 2009; Zelent et al., 2011
**S64Y**	Christesen, Tribble, et al., 2008; Zelent et al., 2011; Langer et al., 2019
**T65I**	Gloyn et al., 2003; Heredia, Carlson, et al., 2006; Christesen, Tribble, et al., 2008; Cullen et al., 2011; Zelent et al., 2011; Martinez et al., 2016; Martinez et al., 2017
G68K	Zelent et al., 2011
**G68V**	Wabitsch et al., 2007‡; Zelent et al., 2011; Langer et al., 2019
S69P	Pal & Miller, 2009
D73E	Pal & Miller, 2009
**K90R**	Ping et al., 2019
**V91L**	Pal & Miller, 2009; Kassem et al., 2010; Zelent et al., 2011; Martinez et al., 2016; Martinez et al., 2017; Lu et al., 2019
**W99C**	Zelent et al., 2008; Martinez et al., 2016; Martinez et al., 2017
**W99L**	Sayed et al., 2009; Snider et al., 2013; Tornovsky-Babeay et al., 2014
**W99R**	Gloyn et al., 2003; Heredia, Carlson, et al., 2006; Christesen, Tribble, et al., 2008; Zelent et al., 2008; Cullen et al., 2011; Snider et al., 2013
**T103S**	Beer et al., 2011
D158A	Davis et al., 1999
N166R	Moukil et al., 2000
**N180D**	Pal & Miller, 2009; Jannin et al., 2018‡
M197A	Sayed et al., 2009
**M197I**	Sayed et al., 2009; Zelent et al., 2011; Snider et al., 2013
M197L	Sayed et al., 2009; Zelent et al., 2011
**M197T**	Sayed et al., 2009; Morishita et al., 2017
**M197V**	Pal & Miller, 2009; Sayed et al., 2009; Ping et al., 2019‡
**I211F**†	Pal & Miller, 2009; Henquin et al., 2013
Y214A	Moukil et al., 2000; Heredia, Carlson, et al., 2006; Zelent et al., 2011
**Y214C**	Cuesta-Munoz et al., 2004; Pedelini et al., 2005; Heredia, Carlson, et al., 2006; Heredia, Thomson, et al., 2006; Cullen et al., 2011; Zelent et al., 2011; Snider et al., 2013; Tornovsky-Babeay et al., 2014
Y215A	Pedelini et al., 2005; Heredia, Carlson, et al., 2006; Zelent et al., 2011
E216D	Pal & Miller, 2009
W257F	Zelent et al., 2008
K296M	Moukil et al., 2000
**V389L**	Beer et al., 2011; Zelent et al., 2011; Snider et al., 2013; Challis et al., 2014‡
**E442K**	Barbetti et al., 2009; Zelent et al., 2011; Martinez et al., 2016; Martinez et al., 2017
G446S	Pal & Miller, 2009
**V452L**	Cuesta-Munoz et al., 2008; Meissner et al., 2009; Zelent et al., 2011; Ajala et al., 2016‡
S453A	Pal & Miller, 2009
**ins454A**	Sayed et al., 2009; Zelent et al., 2011; Snider et al., 2013; Tornovsky-Babeay et al., 2014; Tornovsky-Babeay et al., 2021
**V455L**	Hillebrand et al., 2009; Lange et al., 2011
**V455M**	Glaser et al., 1998‡; Burke et al., 1999; Davis et al., 1999; Heredia, Carlson, et al., 2006; Cullen et al., 2011; Zelent et al., 2011; Tornovsky-Babeay et al., 2014‡; Maiorana et al., 2015‡
**A456V**	Christesen et al., 2002‡; Dullaart et al., 2004‡; Pedelini et al., 2005; Heredia, Carlson, et al., 2006; Pino et al., 2007; Christesen, Brusgaard, et al., 2008‡; Christesen, Tribble, et al., 2008; Cullen et al., 2011; Vidal-Alabro et al., 2011; Zelent et al., 2011
A460R	Pedelini et al., 2005

ESM Table 1. Overview of published GK variants that display enhanced enzyme activity, including in vitro kinetic studies. To date, 22 of these variants have been described in individuals diagnosed with hyperinsulinaemic hypoglycaemia. These are listed in bold and the reference publications are underlined. *Mutant V62M activates in vitro, but paradoxically results in a MODY2 phenotype; †mutant I211F was somatic, described in one individual in pathological islets only; ‡these publications reported overweight in affected individuals. The reference details are as follows:

Ajala, O. N., Huffman, D. M., & Ghobrial, II. (2016). Glucokinase mutation-a rare cause of recurrent hypoglycemia in adults: a case report and literature review. *J Community Hosp Intern Med Perspect, 6*(5), 32983. doi:10.3402/jchimp.v6.32983

Barbetti, F., Cobo-Vuilleumier, N., Dionisi-Vici, C., Toni, S., Ciampalini, P., Massa, O., Rodriguez-Bada, P., Colombo, C., Lenzi, L., Garcia-Gimeno, M. A., Bermudez-Silva, F. J., Rodriguez de Fonseca, F., Banin, P., Aledo, J. C., Baixeras, E., Sanz, P., & Cuesta-Munoz, A. L. (2009). Opposite clinical phenotypes of glucokinase disease: Description of a novel activating mutation and contiguous inactivating mutations in human glucokinase (GCK) gene. *Mol Endocrinol, 23*(12), 1983-1989. doi:10.1210/me.2009-0094

Beer, N. L., van de Bunt, M., Colclough, K., Lukacs, C., Arundel, P., Chik, C. L., Grimsby, J., Ellard, S., & Gloyn, A. L. (2011). Discovery of a novel site regulating glucokinase activity following characterization of a new mutation causing hyperinsulinemic hypoglycemia in humans. *J Biol Chem, 286*(21), 19118-19126. doi:10.1074/jbc.M111.223362

Burke, C. V., Buettger, C. W., Davis, E. A., McClane, S. J., Matschinsky, F. M., & Raper, S. E. (1999). Cell-biological assessment of human glucokinase mutants causing maturity-onset diabetes of the young type 2 (MODY-2) or glucokinase-linked hyperinsulinaemia (GK-HI). *Biochem J, 342 (Pt 2)*, 345-352.

Challis, B. G., Harris, J., Sleigh, A., Isaac, I., Orme, S. M., Seevaratnam, N., Dhatariya, K., Simpson, H. L., & Semple, R. K. (2014). Familial adult onset hyperinsulinism due to an activating glucokinase mutation: implications for pharmacological glucokinase activation. *Clin Endocrinol (Oxf), 81*(6), 855-861. doi:10.1111/cen.12517

Christesen, H. B., Brusgaard, K., Beck Nielsen, H., & Brock Jacobsen, B. (2008). Non-insulinoma persistent hyperinsulinaemic hypoglycaemia caused by an activating glucokinase mutation: hypoglycaemia unawareness and attacks. *Clin Endocrinol (Oxf), 68*(5), 747-755. doi:10.1111/j.1365-2265.2008.03184.x

Christesen, H. B., Jacobsen, B. B., Odili, S., Buettger, C., Cuesta-Munoz, A., Hansen, T., Brusgaard, K., Massa, O., Magnuson, M. A., Shiota, C., Matschinsky, F. M., & Barbetti, F. (2002). The second activating glucokinase mutation (A456V): implications for glucose homeostasis and diabetes therapy. *Diabetes, 51*(4), 1240-1246. doi:10.2337/diabetes.51.4.1240

Christesen, H. B., Tribble, N. D., Molven, A., Siddiqui, J., Sandal, T., Brusgaard, K., Ellard, S., Njolstad, P. R., Alm, J., Brock Jacobsen, B., Hussain, K., & Gloyn, A. L. (2008). Activating glucokinase (GCK) mutations as a cause of medically responsive

congenital hyperinsulinism: prevalence in children and characterisation of a novel GCK mutation. *Eur J Endocrinol, 159*(1), 27-34. doi:10.1530/EJE-08-0203

Cuesta-Munoz, A. L., Huopio, H., Otonkoski, T., Gomez-Zumaquero, J. M., Nanto-Salonen, K., Rahier, J., Lopez-Enriquez, S., Garcia-Gimeno, M. A., Sanz, P., Soriguer, F. C., & Laakso, M. (2004). Severe persistent hyperinsulinemic hypoglycemia due to a de novo glucokinase mutation. *Diabetes, 53*(8), 2164-2168. doi:10.2337/diabetes.53.8.2164

Cuesta-Munoz, A. L., Meissner, T., Cobo-Vuilleumier, N., Maringa, M., Garcia-Gimeno, M. A., Castro-Santiago, M. J., Aledo, J. C., Weber, J., & Sanz, P. (2008). The second "de novo" activating glucokinase mutation (V452L) in a patient with developmental delay. *Diabetologia, 51*, S123-S123.

Cullen, K. S., Matschinsky, F. M., Agius, L., & Arden, C. (2011). Susceptibility of glucokinase-MODY mutants to inactivation by oxidative stress in pancreatic beta-cells. *Diabetes, 60*(12), 3175-3185. doi:10.2337/db11-0423

Davis, E. A., Cuesta-Munoz, A., Raoul, M., Buettger, C., Sweet, I., Moates, M., Magnuson, M. A., & Matschinsky, F. M. (1999). Mutants of glucokinase cause hypoglycaemia- and hyperglycaemia syndromes and their analysis illuminates fundamental quantitative concepts of glucose homeostasis. *Diabetologia, 42*(10), 1175-1186. doi:10.1007/s001250051289

Dullaart, R. P., Hoogenberg, K., Rouwe, C. W., & Stulp, B. K. (2004). Family with autosomal dominant hyperinsulinism associated with A456V mutation in the glucokinase gene. *J Intern Med, 255*(1), 143-145. doi:10.1046/j.0954-6820.2003.01243.x

Glaser, B., Kesavan, P., Heyman, M., Davis, E., Cuesta, A., Buchs, A., Stanley, C. A., Thornton, P. S., Permutt, M. A., Matschinsky, F. M., & Herold, K. C. (1998). Familial hyperinsulinism caused by an activating glucokinase mutation. *N Engl J Med, 338*(4), 226-230. doi:10.1056/NEJM199801223380404

Gloyn, A. L., Noordam, K., Willemsen, M. A., Ellard, S., Lam, W. W., Campbell, I. W., Midgley, P., Shiota, C., Buettger, C., Magnuson, M. A., Matschinsky, F. M., & Hattersley, A. T. (2003). Insights into the biochemical and genetic basis of glucokinase activation from naturally occurring hypoglycemia mutations. *Diabetes, 52*(9), 2433-2440. doi:10.2337/diabetes.52.9.2433

Gloyn, A. L., Odili, S., Zelent, D., Buettger, C., Castleden, H. A., Steele, A. M., Stride, A., Shiota, C., Magnuson, M. A., Lorini, R., d'Annunzio, G., Stanley, C. A., Kwagh, J., van Schaftingen, E., Veiga-da-Cunha, M., Barbetti, F., Dunten, P., Han, Y., Grimsby, J., Taub, R., Ellard, S., Hattersley, A. T., & Matschinsky, F. M. (2005). Insights into the structure and regulation of glucokinase from a novel mutation (V62M), which causes maturity-onset diabetes of the young. *J Biol Chem, 280*(14), 14105-14113. doi:10.1074/jbc.M413146200

Henquin, J. C., Sempoux, C., Marchandise, J., Godecharles, S., Guiot, Y., Nenquin, M., & Rahier, J. (2013). Congenital hyperinsulinism caused by hexokinase I expression or glucokinase-activating mutation in a subset of beta-cells. *Diabetes, 62*(5), 1689-1696. doi:10.2337/db12-1414

Heredia, V. V., Carlson, T. J., Garcia, E., & Sun, S. (2006). Biochemical basis of glucokinase activation and the regulation by glucokinase regulatory protein in naturally occurring mutations. *J Biol Chem, 281*(52), 40201-40207. doi:10.1074/jbc.M607987200

Heredia, V. V., Thomson, J., Nettleton, D., & Sun, S. (2006). Glucose-induced conformational changes in glucokinase mediate allosteric regulation: transient kinetic analysis. *Biochemistry, 45*(24), 7553-7562. doi:10.1021/bi060253q

Hillebrand, G., Tsiakas, K., Bergmann, J., Witsch, M., Ullrich, K., & Santer, R. (2009). Infantile hyperinsulinismus and adult type-2 diabetes in carriers of activating glucokinase gene (*GCK*)-mutations. *Molecular Genetics and Metabolism, 98*(1-2), 45-45.

Jannin, A., Espiard, S., Douillard, C., Pasquier, F., Bellanne-Chantelot, C., & Vantyghem, M. C. (2018). Hyperinsulinemic hypoglycemia without insulinoma: Think of activating glucokinase mutation. *Presse Med, 47*(6), 595-597. doi:10.1016/j.lpm.2018.02.015

Kassem, S., Bhandari, S., Rodriguez-Bada, P., Motaghedi, R., Heyman, M., Garcia-Gimeno, M. A., Cobo-Vuilleumier, N., Sanz, P., Maclaren, N. K., Rahier, J., Glaser, B., & Cuesta-Munoz, A. L. (2010). Large islets, beta-cell proliferation, and a glucokinase mutation. *N Engl J Med, 362*(14), 1348-1350. doi:10.1056/NEJMc0909845

Lange, A., Szalapska, M., Starostecka, E., Lewinski, A., Grodzicka, A., & Gulczynska, E. (2011). Congenital hyperinsulinemic hypoglycemia (HH) as a result of glucokinase mutation - case report. *J Inherit Metab Dis, 34*(Suppl 3), 174.

Langer, S., Hofmeister-Brix, A., Waterstradt, R., & Baltrusch, S. (2019). 6-Phosphofructo-2-kinase/fructose-2,6-bisphosphatase and small chemical activators affect enzyme activity of activating glucokinase mutants by distinct mechanisms. *Biochem Pharmacol, 168*, 149-161. doi:10.1016/j.bcp.2019.06.024

Lu, B., Tonne, J. M., Munoz-Gomez, M., & Ikeda, Y. (2019). Hyperinsulinemic hypoglycemia subtype glucokinase V91L mutant induces necrosis in beta-cells via ATP depletion. *Biochem Biophys Rep, 17*, 108-113. doi:10.1016/j.bbrep.2018.12.002

Maiorana, A., Manganozzi, L., Barbetti, F., Bernabei, S., Gallo, G., Cusmai, R., Caviglia, S., & Dionisi-Vici, C. (2015). Ketogenic diet in a patient with congenital hyperinsulinism: a novel approach to prevent brain damage. *Orphanet J Rare Dis, 10*, 120. doi:10.1186/s13023-015-0342-6

Martinez, R., Fernandez-Ramos, C., Vela, A., Velayos, T., Aguayo, A., Urrutia, I., Rica, I., Castano, L., & Spanish Congenital Hyperinsulinism, G. (2016). Clinical and genetic characterization of congenital hyperinsulinism in Spain. *Eur J Endocrinol, 174*(6), 717-726. doi:10.1530/EJE-16-0027

Martinez, R., Gutierrez-Nogues, A., Fernandez-Ramos, C., Velayos, T., Vela, A., Spanish Congenital Hyperinsulinism, G., Navas, M. A., & Castano, L. (2017). Heterogeneity in phenotype of hyperinsulinism caused by activating glucokinase mutations: a novel mutation and its functional characterization. *Clin Endocrinol (Oxf), 86*(6), 778-783. doi:10.1111/cen.13318

Meissner, T., Marquard, J., Cobo-Vuilleumier, N., Maringa, M., Rodriguez-Bada, P., Garcia-Gimeno, M. A., Baixeras, E., Weber, J., Olek, K., Sanz, P., Mayatepek, E., & Cuesta-Munoz, A. L. (2009). Diagnostic difficulties in glucokinase hyperinsulinism. *Horm Metab Res, 41*(4), 320-326. doi:10.1055/s-0028-1102922

Morishita, K., Kyo, C., Yonemoto, T., Kosugi, R., Ogawa, T., & Inoue, T. (2017). Asymptomatic congenital hyperinsulinism due to a glucokinase-activating mutation, treated as adrenal insufficiency for twelve years. *Case Rep Endocrinol, 2017*, 4709262. doi:10.1155/2017/4709262

Moukil, M. A., Veiga-da-Cunha, M., & Van Schaftingen, E. (2000). Study of the regulatory properties of glucokinase by site-directed mutagenesis: conversion of glucokinase to an enzyme with high affinity for glucose. *Diabetes, 49*(2), 195-201. doi:10.2337/diabetes.49.2.195

Pal, P., & Miller, B. G. (2009). Activating mutations in the human glucokinase gene revealed by genetic selection. *Biochemistry, 48*(5), 814-816. doi:10.1021/bi802142q

Pedelini, L., Garcia-Gimeno, M. A., Marina, A., Gomez-Zumaquero, J. M., Rodriguez-Bada, P., Lopez-Enriquez, S., Soriguer, F. C., Cuesta-Munoz, A. L., & Sanz, P. (2005).

Structure-function analysis of the alpha5 and the alpha13 helices of human glucokinase: description of two novel activating mutations. *Protein Sci, 14*(8), 2080-2086. doi:10.1110/ps.051485205

Ping, F., Wang, Z., & Xiao, X. (2019). Clinical and enzymatic phenotypes in congenital hyperinsulinemic hypoglycemia due to glucokinase-activating mutations: A report of two cases and a brief overview of the literature. *J Diabetes Investig, 10*(6), 1454-1462. doi:10.1111/jdi.13072

Pino, M. F., Kim, K. A., Shelton, K. D., Lindner, J., Odili, S., Li, C., Collins, H. W., Shiota, M., Matschinsky, F. M., & Magnuson, M. A. (2007). Glucokinase thermolability and hepatic regulatory protein binding are essential factors for predicting the blood glucose phenotype of missense mutations. *J Biol Chem, 282*(18), 13906-13916. doi:10.1074/jbc.M610094200

Sayed, S., Langdon, D. R., Odili, S., Chen, P., Buettger, C., Schiffman, A. B., Suchi, M., Taub, R., Grimsby, J., Matschinsky, F. M., & Stanley, C. A. (2009). Extremes of clinical and enzymatic phenotypes in children with hyperinsulinism caused by glucokinase activating mutations. *Diabetes, 58*(6), 1419-1427. doi:10.2337/db08-1792

Snider, K. E., Becker, S., Boyajian, L., Shyng, S. L., MacMullen, C., Hughes, N., Ganapathy, K., Bhatti, T., Stanley, C. A., & Ganguly, A. (2013). Genotype and phenotype correlations in 417 children with congenital hyperinsulinism. *J Clin Endocrinol Metab, 98*(2), E355-363. doi:10.1210/jc.2012-2169

Tornovsky-Babeay, S., Dadon, D., Ziv, O., Tzipilevich, E., Kadosh, T., Schyr-Ben Haroush, R., Hija, A., Stolovich-Rain, M., Furth-Lavi, J., Granot, Z., Porat, S., Philipson, L. H., Herold, K. C., Bhatti, T. R., Stanley, C., Ashcroft, F. M., In't Veld, P., Saada, A., Magnuson, M. A., Glaser, B., & Dor, Y. (2014). Type 2 diabetes and congenital hyperinsulinism cause DNA double-strand breaks and p53 activity in beta cells. *Cell Metab, 19*(1), 109-121. doi:10.1016/j.cmet.2013.11.007

Tornovsky-Babeay, S., Weinberg-Corem, N., Ben-Haroush Schyr, R., Avrahami, D., Lavi, J., Feleke, E., Kaestner, K. H., Dor, Y., & Glaser, B. (2021). Biphasic dynamics of beta cell mass in a mouse model of congenital hyperinsulinism: implications for type 2 diabetes. *Diabetologia, 64*(5), 1133-1143. doi:10.1007/s00125-021-05390-x

Vidal-Alabro, A., Gomez-Valades, A. G., Mendez-Lucas, A., Llorens, J., Bartrons, R., Bermudez, J., & Perales, J. C. (2011). Liver glucokinase(A456V) induces potent hypoglycemia without dyslipidemia through a paradoxical induction of the catalytic subunit of glucose-6-phosphatase. *Int J Endocrinol, 2011*, 707928. doi:10.1155/2011/707928

Wabitsch, M., Lahr, G., Van de Bunt, M., Marchant, C., Lindner, M., von Puttkamer, J., Fenneberg, A., Debatin, K. M., Klein, R., Ellard, S., Clark, A., & Gloyn, A. L. (2007). Heterogeneity in disease severity in a family with a novel G68V GCK activating mutation causing persistent hyperinsulinaemic hypoglycaemia of infancy. *Diabet Med, 24*(12), 1393-1399. doi:10.1111/j.1464-5491.2007.02285.x

Zelent, B., Odili, S., Buettger, C., Shiota, C., Grimsby, J., Taub, R., Magnuson, M. A., Vanderkooi, J. M., & Matschinsky, F. M. (2008). Sugar binding to recombinant wild-type and mutant glucokinase monitored by kinetic measurement and tryptophan fluorescence. *Biochem J, 413*(2), 269-280. doi:10.1042/BJ20071718

Zelent, B., Odili, S., Buettger, C., Zelent, D. K., Chen, P., Fenner, D., Bass, J., Stanley, C., Laberge, M., Vanderkooi, J. M., Sarabu, R., Grimsby, J., & Matschinsky, F. M. (2011). Mutational analysis of allosteric activation and inhibition of glucokinase. Biochem J, 440(2), 203-215. doi:10.1042/BJ20110440

ESM Table 2

	Patient II-2	Patient II-5	Patient III-4
Triglycerides (mmol/l) [0.80-2.05]	2.45	0.67	0.24
Total cholesterol (mmol/l) [3.90-6.24]	5.46	4.03	4.11

ESM Table 2. Fasting serum triglyceride and cholesterol levels in patients II-2, II-5, and III-4.

ESM Figure 1

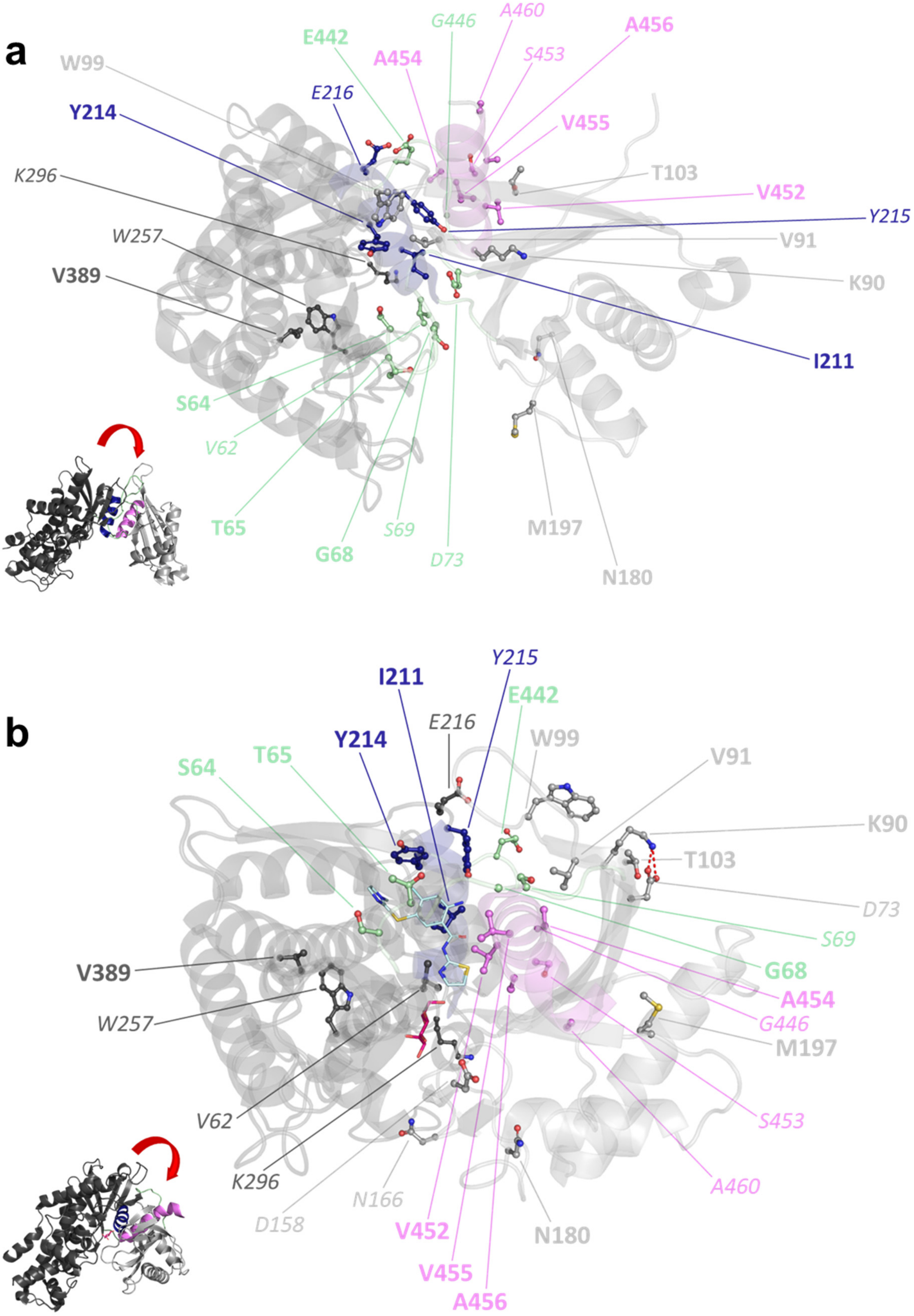

a
W99
E442
G446
A460
A456
A454
S453
E216
Y214
V455
K296
T103
V452
Y215
W257
V91
V389
K90
I211
S64
V62
T65
S69
G68
D73
M197
N180
b
I211
Y215
E442
E216
Y214
W99
T65
S64
V91
K90
T103
D73
S69
V389
G68
A454
W257
G446
M197
V62
S453
K296
A460
N166
D158
V452
N180
V455
A456

ESM Figure 1. Intramolecular locations of GK amino acids for which activating mutations have been described. The 29 residues of wild-type GK, as listed in ESM Table 1, are highlighted as ball-and-stick models (alpha-carbons plus side chains, with oxygen red, nitrogen blue, sulphur yellow) within the structures of the super-open (a, 1v4t [1]) and the closed conformation of GK (b, 1v4s [1]). The molecule view shows the allosteric site, with the large domain in dark grey and the small domain in light grey, the interconnecting regions I-III in pale green, the C-terminal helix α13 in violet, and helix α5 in dark blue. The labelling of the highlighted residues also follows this colour scheme. Italic labelling indicates that activating mutations of the particular amino acid have not to date been found in humans. In (b), glucose is shown in magenta, the GKA compound A (2-amino-4-fluoro-5-[(1-methyl-1*H*-imidazol-2-yl)sulfanyl]-*N*-(1,3-thiazol-2-yl)benzamide) in cyan, and a hydrogen bond between residues Asp-73 and Lys-90 – suggesting a similar mechanism of activation for the variants D73E and K90R – as red dotted lines. Note that residues Asp-158 and Asn-166 are not displayed in the super-open GK conformation (a) because they lie within a mobile loop structure of the small domain that is disordered in the unliganded state. Also note that the secondary structure of the protein varies slightly between the super-open and the closed enzyme conformation: for example, residue Gly-446 is part of interconnecting region III in (a) but part of helix α13 in (b).

[1] Kamata K, Mitsuya M, Nishimura T, Eiki J, Nagata Y (2004) Structural basis for allosteric regulation of the monomeric allosteric enzyme human glucokinase. Structure 12(3): 429-438. 10.1016/j.str.2004.02.005

ESM Figure 2

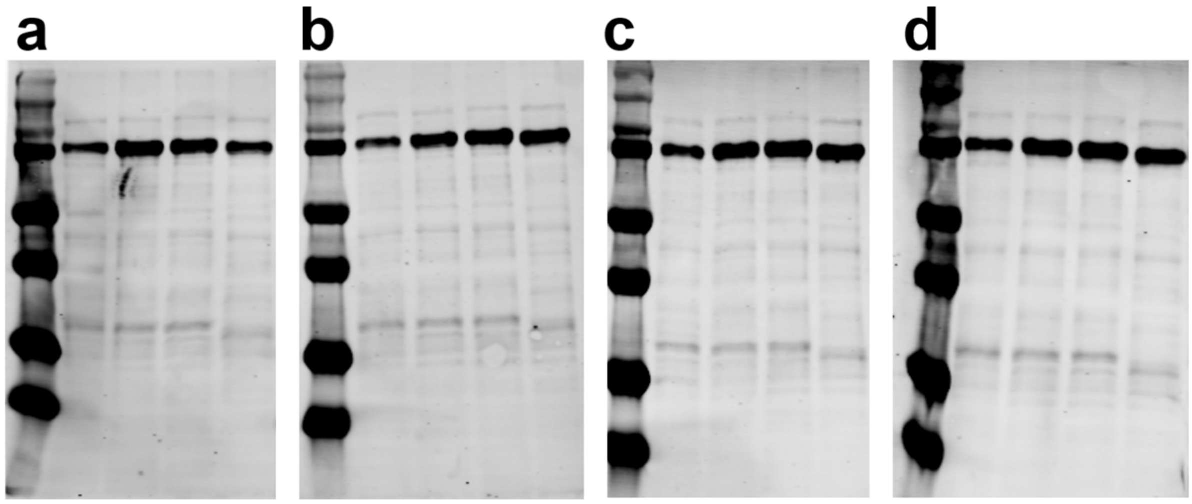

ESM Figure 2. GK Western blot analyses of recombinant Dendra2-GK wild-type, M455, E455, and L455 enzymes. 5 µg of recombinant protein of each preparation was loaded in a 10% polyacrylamide gel. Representative blots (a – d) of the experiments are shown. Molecular weight marker 20, 25, 37, 50, 75 kDa (lane 1), Dendra2-GK wild-type (lane 2), Dendra2-GK M455 (lane 3), Dendra2-GK E455 (lane 4), and Dendra2-GK L455 (lane 5).

ESM Figure 3

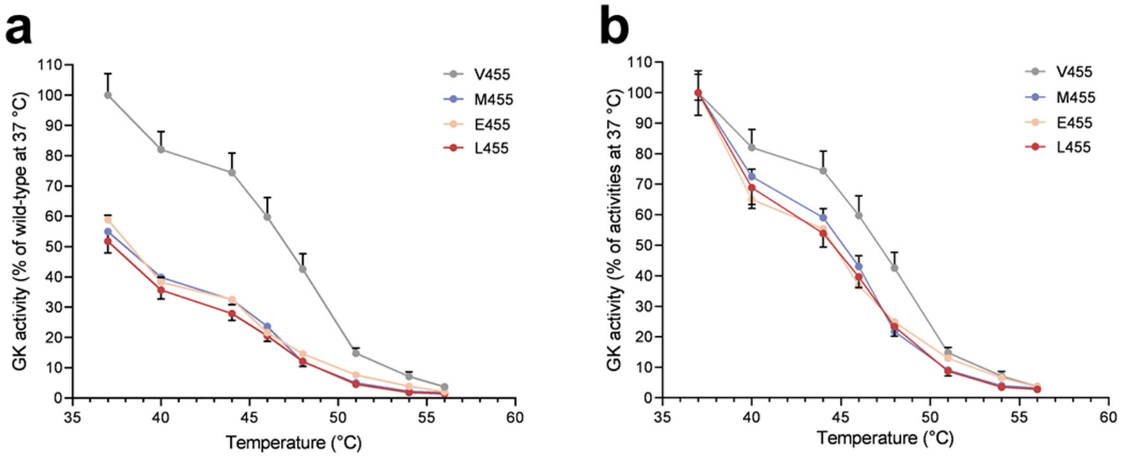

ESM Figure 3. Thermostability of recombinant Dendra2-GK wild-type, -M455, -E455, and -L455 enzymes. Glucose-phosphorylating activities of 1 µg recombinant fusion proteins were measured after 30 min heating at the temperature indicated prior to glucose addition (100 mmol/l). Data are shown as means ± SEM from seven independent experiments, expressed as (a) percentage of wild-type activity after incubation at 37°C or as (b) percentage relative to the respective activities at 37°C.
